# Nanoneedles enable spatiotemporal lipidomics of living tissues

**DOI:** 10.1038/s41565-025-01955-8

**Published:** 2025-06-16

**Authors:** Chenlei Gu, Davide Alessandro Martella, Leor Ariel Rose, Nadia Rouatbi, Cong Wang, Alaa Zam, Valeria Caprettini, Magnus Jensen, Shiyue Liu, Cathleen Hagemann, Siham Memdouh, Andrea Serio, Vincenzo Abbate, Khuloud T. Al-Jamal, Maddy Parsons, Mads S. Bergholt, Paul M. Brennan, Assaf Zaritsky, Ciro Chiappini

**Affiliations:** 1 Centre for Craniofacial and Regenerative Biology, King’s College London, London, UK; 2 London Centre for Nanotechnology, King’s College London, London, UK; 3 Department of Software and Information Systems Engineering, Ben-Gurion University of the Negev, Be’er Sheva, Israel; 4 Institute of Pharmaceutical Science, King’s College London, London, UK; 5 Wenzhou eye valley innovation center, Eye Hospital, Wenzhou Medical University, Zhejiang, China; 6 UK Dementia Research Institute at King’s College London, London, UK; 7 Department of Basic and Clinical Neuroscience, Institute of Psychiatry, Psychology and Neuroscience, King’s College London, London, UK; 8 The Francis Crick Institute, London, UK; 9 Department of Pharmacology and Pharmacy, Li Ka Shing Faculty of Medicine, The University of Hong Kong, Hong Kong, Hong Kong Special Administrative Region, China; 10 Randall Centre for Cell and Molecular Biophysics, King’s College London, London, UK; 11 Translational Neurosurgery, Centre for Clinical Brain Sciences, University of Edinburgh, Edinburgh, UK

**Keywords:** Nanobiotechnology, Diagnostic devices

## Abstract

Spatial biology provides high-content diagnostic information by mapping the molecular composition of tissues. However, traditional spatial biology approaches typically require non-living samples, limiting temporal analysis. Here, to address this limitation, we present a workflow using porous silicon nanoneedles to repeatedly collect biomolecules from live brain tissues and map lipid distribution through desorption electrospray ionization mass spectrometry imaging. This method preserves the integrity of the original tissue while replicating its spatial molecular profile on the nanoneedle substrate, accurately reflecting lipid distribution and tissue morphology. Machine learning analysis of 23 human glioma biopsies demonstrated that nanoneedle sampling enables the precise classification of disease states. Furthermore, a spatiotemporal analysis of mouse gliomas treated with temozolomide revealed time- and treatment-dependent variations in lipid composition. Our approach enables non-destructive spatiotemporal lipidomics, advancing molecular diagnostics for precision medicine.

## Abstract (web summary)

Arrays of silicon nanoneedles are used to generate molecular replicas of live brain tissue for longitudinal spatial lipidomic classification via desorption electrospray ionization mass spectrometry imaging of gliomas and to monitor the responses of the tumours to chemotherapy.

## Main

Spatial biology provides an omics-level understanding of complex molecular landscapes, offering insight into development, regeneration and disease progression^1–3^. In particular, spatial lipidomics gives insight into metabolism and its perturbations during physiological and pathological processes^4,5^. However, spatial biology typically relies on destructive processes in non-living tissues, limiting its applicability for the study of unique samples or to track the spatiotemporal dynamics of living systems.

To address these limitations, vertical nanoprobes, such as nanoneedles, offer a transformative approach to extend spatial biology into the temporal domain by enabling repeated, non-perturbing sampling from the same live specimen. Nanoprobes can access intracellular compartments to collect biomolecules with minimal disruption to cell function^6,7^, enabling repeated or continuous sampling^8–11^. Among them, porous silicon nanoneedles are particularly adept to achieve this vision. They interface with tissue without toxicity^12^ and map biomarkers across tissue with cellular resolution, capturing heterogeneity^13^. Furthermore, mass spectrometry (MS) imaging on porous silicon provides tissue maps with high sensitivity and low interference^14,15^.

Gliomas are an ideal model to test spatiotemporal lipidomics in live tissues^4,5^. Gliomas reprogram lipid metabolism to enhance cell membrane integrity and support abnormal cell proliferation^16^, exploiting aberrant lipid profiles to sustain their growth. Their heterogeneous lipid dysregulation carries relevant information for clinical intervention^17,18^, emphasizing the importance of spatiotemporal lipidomics profiles.

**Fig. 1:**
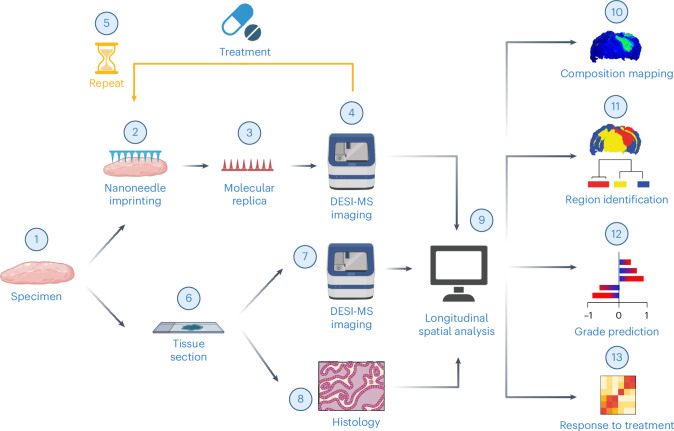
The molecular replica workflow. The process begins with the collection of mouse brains and HG biopsies (1). Nanoneedle imprinting of these specimens (2) generates molecular replicas (3) that are analysed using DESI-MSI (4). Repeated sampling of molecular replicas from the same specimen over time enables study of the evolution of its metabolic profile (5). Specimens can also be processed to produce tissue sections proximal to the molecular replica (6) to obtain molecular (7) and morphological references (8). The analysis of the DESI-MSI datasets (9) provides information on the intensity distribution of key lipids to map the tissue composition (10) as well as the ability to identify tissue regions such as white matter, grey matter and different types of lesions (11) and to determine tissue similarity and grade from HG biopsies (12). The longitudinal analysis provides insights into a tissue’s response to treatment (13). Created with BioRender.com.

Here, we present an integrated workflow for spatiotemporal lipidomics applied to the study of gliomas (Fig. 1). Using nanoneedles, we repeatedly generated non-destructive imprints of brain tissue, called molecular replicas. Desorption electrospray ionization MS imaging (DESI-MSI) analysis of the replicas generated lipidomic maps that accurately captured key brain morphological features, closely matching the species abundance and spatial distribution observed in the original tissue. Molecular replicas matched tissue sections in their performance for grading human glioma (HG) samples on the basis of lipidomic maps. Moreover, longitudinal spatial lipidomic profiling of glioma-bearing brain tissue slices revealed lipid- and sample-specific temporal changes in response to chemotherapy. This approach establishes a minimally perturbative method for spatiotemporal lipidomics of live tissues, with transformative potential for studying tissue dynamics and therapeutic responses.

## Results

### Lipidomics on nanoneedles

**Extended Data Fig. 1:**
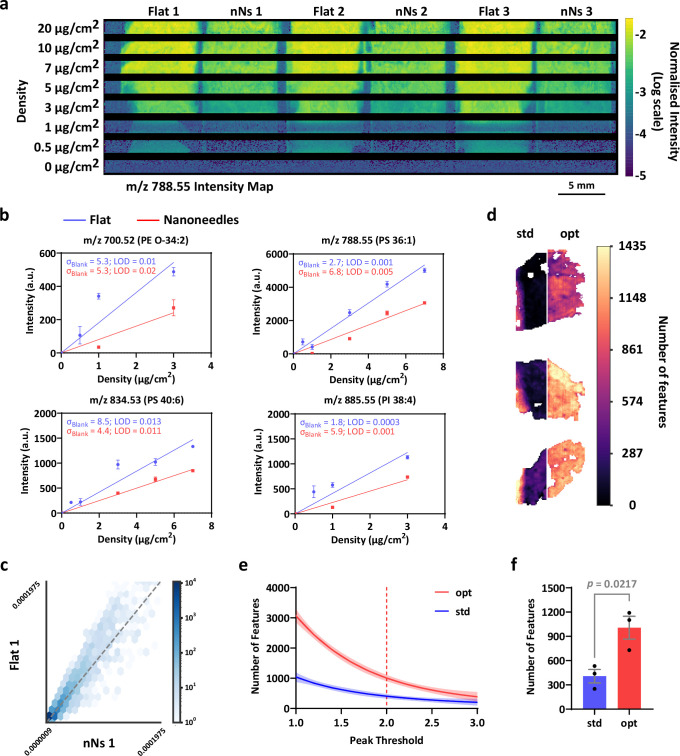
Lipidomics on nanoneedles. (**a**) DESI-MSI intensity maps showing the most intense peak at *m/z* 788.55 (PS 36:1) from porcine brain homogenate drop-deposited onto three flat and three nanoneedle chips, across a density range of 0–20 μg/cm². (**b**) Calibration curves derived from the DESI-MSI of porcine brain homogenate in panel (a), relative to lipid peaks *m/z* 700.52 (PE O-34:2), 788.55 (PS 36:1), 834.53 (PS 40:6), and 885.55 (PI 38:4). The standard deviation of the blank(σ_blank_) and the calculated limit of detection (LOD) are provided. Data: mean ± s.e.m., (N = 3 independent experimental replicates). (**c**) Correlation scatter plot of spectra from flat and nanoneedle surfaces at a concentration of 7 µg/cm², illustrating the relative abundance of all detected species. (**d**-**f**) Optimization of DESI imaging conditions for nanoneedles: (**d**) maps depicting the number of features from three mouse liver replicas on nanoneedles, imaged with standard (std, left) and optimized (opt, right) DESI parameters. (**e**) Quantitative analysis of the number of features as a function of the confidence threshold set for peak identification, under standard (std) and optimized (opt) DESI-MSI conditions. Data: mean ± s.e.m., with 95% confidence and prediction bands from a nonlinear fit (one-phase decay model). (**f**) Bar graph comparing the number of features before (std) and after (opt) the optimization of DESI parameters at a peak threshold value of 2.0, corresponding to the 95% confidence threshold. Data: mean ± s.e.m. (N = 3 independent biological replicates). Statistics: two-tailed unpaired t test. Source data

We assessed the suitability of nanoneedles for DESI-MSI lipidomics, using nanoneedle arrays with 2 μm pitch, 4.00 μm height, 50 nm tip diameter, 600 nm base diameter, 77.2 m^2^ g^−1^ surface area, 50% porosity and 7.9 nm average pore diameter, on 8 × 8 mm silicon substrates (Supplementary Fig. 1). The analysis of porcine brain lipid extract revealed that nanoneedles and flat surfaces offer comparable sensitivity for the most representative lipids, consistent with established DESI limits of detection (LODs)^19^ (Extended Data Fig. 1a,b and Supplementary Table 1). Nanoneedle substrates typically displayed slightly higher LODs than flat silicon, suggesting that nanotextured surfaces can dampen the desorption–ionization process. However, the relative peak intensity correlated well between nanoneedles and flat substrates, indicating that the analysis on nanoneedles accurately recapitulated the lipid composition of the mixture (Extended Data Fig. 1c and Supplementary Fig. 2). Optimization of DESI-MSI analytical parameters^20^ for nanoneedles significantly increased the number of detectable features by a factor of 2.47, from 408 ± 83 to 1,007 ± 141 (Extended Data Fig. 1d–f).

### Lipidomics imaging of molecular replicas

**Fig. 2:**
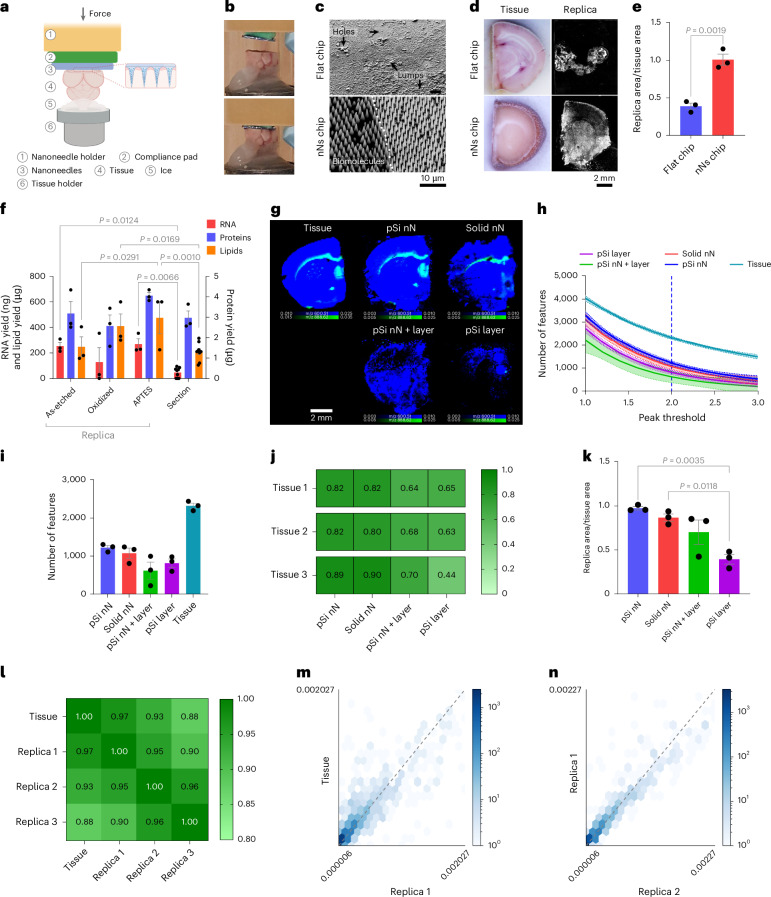
Lipidomics imaging of molecular replicas. **a**, Schematics of the nanoneedle imprinting system. Created with BioRender.com. **b**, Photographs illustrating the system approaching (top) and imprinting (bottom) nanoneedles on tissue. **c**, Scanning electron microscopy images of the molecular adsorbate on flat and nanoneedle substrates after imprinting. **d**, Photographs of the imprinted tissue and fluorescence microscopy images of the corresponding molecular replica. **e**, Quantification of replica-to-tissue area ratio. Data are mean ± s.e.m. (*N* = 3 independent biological replicates). Statistics: two-tailed unpaired *t*-test. **f**, Quantification of RNA, lipids and proteins from nanoneedle replicas with different surface chemistries (as-etched, APTES, oxidized) and a 10-µm brain tissue section. ata are mean ± s.e.m. (*N* = 3 independent biological replicates). Statistics: two-way ANOVA with a post-hoc Tukey’s multiple-comparisons test. **g**, DESI-MSI maps of grey matter (*m*/*z* 834.53, blue) and white matter (*m*/*z* 888.62, green) lipids from a tissue slice and molecular replicas generated using porous silicon nanoneedles (pSi nN), solid silicon nanoneedles (solid nN), porous silicon nanoneedles over a porous silicon layer (pSi nN + layer) and a porous silicon layer (pSi layer). **h**, Quantification of the number of features as a function of the confidence threshold set for peak identification in samples from **g**. Data are mean ± s.e.m. (*N* = 3 independent biological replicates). **i**, The feature count at a peak threshold of 2.0 (95% confidence) for samples from **g**. Data are mean ± s.e.m. (*N* = 3 independent biological replicates). **j**, The correlation matrix of spectra from three tissue sections and their corresponding replicas from **g**. **k**, The relative surface area of molecular replicas to original tissue for samples from **g**. Data are mean ± s.e.m. (*N* = 3 independent biological replicates). Statistics: ordinary one-way ANOVA with post-hoc Tukey’s multiple-comparisons test. **l**, A correlation matrix comparing three pSi nN replicas with the original tissue section. **m**, A correlation scatter plot of lipids peak intensities between the tissue section and the first replica. **n**, A correlation scatter plot of lipid peak intensities between the first and second replica. Source data

We then established the ability of nanoneedles to generate a molecular replica by imprinting frozen mouse brain (Fig. 2a,b). Computational modelling indicated that, during imprinting, a few micrometres of tissue transiently thaw, allowing collection of biomolecules (Supplementary Fig. 3). The resulting replica formed a uniform molecular adsorbate on the nanoneedles, whereas using a flat substrate yielded poor adsorbate uniformity, presenting areas without coverage and areas of adsorbate accumulation (Fig. 2c). The nanoneedle replica fully reproduced the original tissue and macroscopically retained its morphology, whereas the flat replica reproduced only less than half of the tissue, lacking clear morphology (Fig. 2d,e).

We then evaluated the composition of the molecular adsorbate on replicas generated from nanoneedles as-etched, O_2_-plasma oxidized and (3-aminopropyl)-triethoxysilane (APTES) functionalized, assessing the role of surface polarity and charge on biomolecular collection efficiency^21,22^. Oxidation and APTES respectively generated negative and positive charges, providing more polar surfaces than the as-etched porous silicon. For all the functionalizations considered, the amount of collected RNA, proteins and lipids compared favourably with those that could be extracted from the reference tissue (Fig. 2f). RNA collection ranged between 210 ng and 350 ng, while protein collection ranged between 2.5 μg and 4.3 μg. Lipid collection was more efficient on APTES and oxidized nanoneedles compared with as-etched ones, ranging between 200 μg and 600 μg. These data indicated that plasma oxidized nanoneedles were suitable for spatial lipidomics, because their lipid collection efficiency of 410 ± 95 μg was well within the range in which a large number of features were detectable (Fig. 2f) and compared favourably with a 10-μm tissue section (210 ± 23 μg), known to be imageable by DESI-MSI. The use of oxidized nanoneedles also mitigated potential concerns regarding interference from the desorption of polymerized APTES.

We also compared the quality of the replica generated using porous silicon nanoneedles (pSi nN) with those obtained using solid silicon nanoneedles (solid nN), porous silicon nanoneedles over a continuous porous silicon layer (pSi nN + layer) and a continuous porous silicon layer (pSi layer)^23–25^ (Supplementary Fig. 4). pSi nN displayed the largest number of features and the least variability, followed by the solid nN, while the pSi nN + layer and pSi layer yielded less features with higher variability (Fig. 2g–i). The pSi nN also outperformed the other substrates in their spectral correlation to the original tissue, closely matched by solid nN (Fig. 2j), and most closely reproduced the surface area of the original tissue (Fig. 2g,k).

The imprinting was reproducible, with three replicas from the same surface preserving morphological features and showing strong correlation with the reference section and with each other across the whole range from low- to high-abundance lipids (Fig. 2l–n and Supplementary Fig. 5).

These data indicated that a molecular replica generated using porous silicon nanoneedles could accurately and robustly reproduce the lipidomic profile of a tissue. However, the presence of a continuous porous layer reduced the efficiency of the desorption–ionization process, favouring the use of substrates with nanoneedles only. In addition, porous nanoneedles marginally outperformed solid ones, producing replicas that more closely represented the original tissue in terms of area, number of features and correlation.

### Spatial lipidomics of murine glioma replicas

**Fig. 3:**
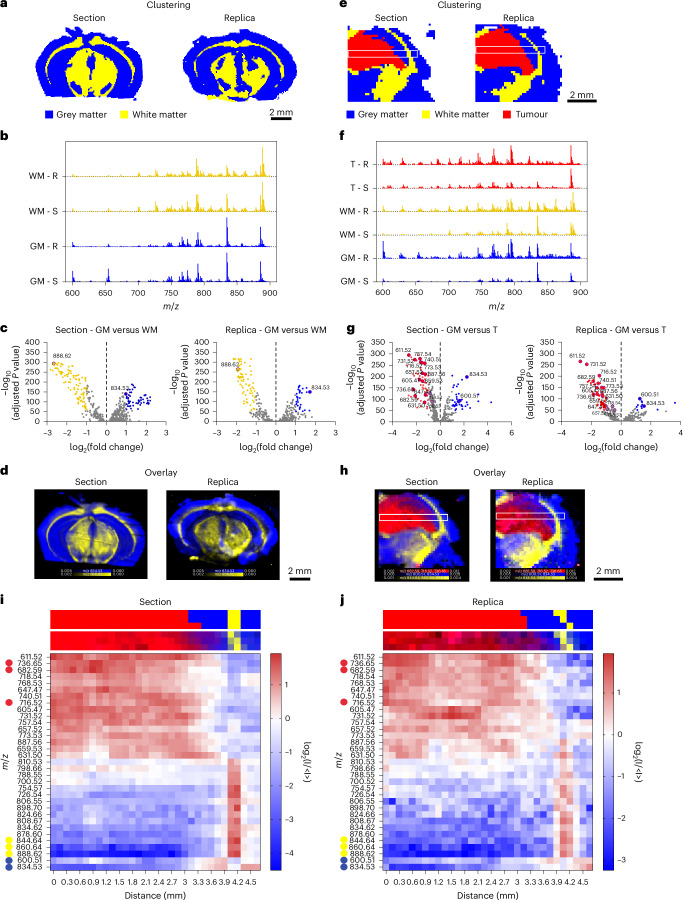
Spatial lipidomics of murine glioma replicas. **a**, HCA maps of the top two clusters from the DESI-MSI data of a murine brain section and its molecular replica, corresponding to grey matter (blue) and white matter (yellow). **b**, Average spectra displaying normalized counts versus *m*/*z*, for the top two clusters associated with grey matter (GM) and white matter (WM) in section (S) and replica (R). **c**, Volcano plots of the differential lipid distribution between grey and white matter, highlighting the role of *m*/*z* 834.53 (PS 40:6) and 888.62 (SHexCer 42:2;O2) as grey and white matter biomarkers in both section and replica. **d**, An overlay map of TIC-normalized intensity for *m*/*z* 834.53 (blue) and 888.62 (yellow). **e**, HCA maps of the top three clusters from the DESI-MSI dataset of a tumour-bearing mouse brain section and replica, corresponding to grey matter (GM, blue), white matter (WM, yellow), and tumour (T, red). White boxes indicate regions used for lipid abundance analysis. **f**, Average spectra showing normalized counts versus *m*/*z*, for the three clusters associated with grey matter (GM), white matter (WM) and tumour (T) in section (S) and replica (R). **g**, Volcano plots comparing lipid distribution between GM and T, highlighting the most representative lipid peaks in both section and replica. **h**, An overlay map of cumulative TIC-normalized intensity for peaks marking grey matter (*m*/*z* 834.53 and *m*/*z* 600.51, blue), white matter (*m*/*z* 844.64, *m*/*z* 860.64 and *m*/*z* 888.62, yellow) and tumour (*m*/*z* 682.59, *m*/*z* 716.52 and *m*/*z* 736.65, red) in section and replica. The white boxes indicate regions used for lipid abundance analysis across GM, WM and T. **i**,**j**, Heatmaps showing the relative abundance of 33 lipid peaks (from analysis in **g**) along the major axis of the white box in **e** and **h** for the section (**i**) and replica (**j**). Top and bottom strips above each heatmap show the cluster assignment and relative lipid intensity for corresponding pixels from **e** and **h**. Source data

We compared the quality of lipidomic imaging on molecular replicas with its proximal tissue section^5^. Hierarchical cluster analysis (HCA) revealed that the top two clusters of both section and replica appeared to align with the areas of grey and white matter within the mouse brain architecture (Fig. 3a). The white matter cluster aligned with brain regions containing highly myelinated fibres, matching the expression pattern of myelin genes such as myelin basic protein in areas such as the corpus callosum. Meanwhile the grey matter cluster corresponded to regions with higher cell body densities and fewer myelinated fibres, such as the cortex^4^.

The lipid composition of these two clusters confirmed this morphological classification (Fig. 3b,c). The higher abundance of *m*/*z* 888.62 (sulfated hexosylceramide (SHexCer) 42:2;O2) and lower abundance of *m/z* 834.53 (phosphatidylserine (PS) 40:6) in white matter were consistent with established DESI-MSI brain biomarkers^26^. Mapping the relative intensity of these two peaks across the sample accurately delineated white and grey matter regions in both the replica and the reference section (Fig. 3d). The congruent distribution of these lipid species across replica and section confirmed the feasibility of generating a lipidomic profile map from a nanoneedle replica of the mouse brain. Minor discrepancies in brain morphology were attributed to the inherent anatomical differences between adjacent tissue sections.

We then used the molecular replica to characterize a cancerous lesion within a mouse glioma^5^. The top three HCA clusters classified white matter, grey matter and tumour regions with comparable effectiveness in section and replica (Fig. 3e). When analysing a dataset including all spectra across tissue and replica, HCA similarly discriminated tumour, white matter and grey matter, highlighting the correspondence of the lipid profile on replica and section. Principal component analysis of intercluster variance further confirmed that both the replica and the section displayed similar characteristic features, enabling accurate mapping of tumour margins and transitions between tissue types (Supplementary Fig. 6).

Comparative analysis of the average mass spectra for each cluster allowed the lipid compositions of white matter, grey matter and tumour to be distinguished and the most relevant differentially abundant species to be identified (Fig. 3f,g and Supplementary Fig. 7). Again, clusters associated with white matter in both the section and replica exhibited a prominent peak at *m*/*z* 888.62 (SHexCer 42:2;O2), while the grey matter clusters showed a prominent peak at *m*/*z* 834.53 (PS 40:6). The tumour cluster showed an increase in the abundance of *m*/*z* 736.65 (ceramide (Cer) 46:1;O4), *m*/*z* 682.59 (Cer 42:0;O4) and *m*/*z* 716.52 (phosphatidylethanolamine (PE) 34:1). The intensity map for these characteristic species effectively discriminated tumour, white matter and grey matter in the replica as well as in the section (Fig. 3h), demonstrating the replica’s ability to accurately reproduce the lipidomic map of the original tissue, even in the presence of a cancerous lesion. The analysis of two additional, distinct brain samples confirmed the reproducibility of our approach (Supplementary Fig. 8).

We assessed the spatial distribution of the 33 most representative species in the section and replica by evaluating their abundance along the major axis (length) of the white box (Fig. 3h and Supplementary Fig. 9). The heatmaps from both section and replica revealed a clear and congruent pattern in lipid abundance across tumour, grey matter and white matter (Fig. 3i,j and Supplementary Fig. 9). The tumour–grey matter transition occurred at 3.1 mm from the left edge. The tumour was rich in lipids at *m*/*z* 736.65 (Cer 46:1;O4), *m*/*z* 682.59 (Cer 42:0;O4) and *m/z* 740.51 (phosphatidylcholine (PC) 30:0), while grey matter showed high levels of *m*/*z* 600.51 (Cer 36:1;O2) and *m*/*z* 834.53 (PS 40:6). At 4 mm, a 0.3 mm white matter layer was enriched in *m*/*z* 844.64 (PS O-41:1), *m*/*z* 860.64 (PS 41:0) and *m*/*z* 888.62 (SHexCer 42:2;O2) followed by grey matter (4.3–4.7 mm) with a lipid profile consistent with the previous grey matter region.

These data indicate that a molecular replica can map the biomolecular architecture of a tumour-bearing tissue. The quality of the replica information enables unsupervised classification of individual pixels with comparable fidelity to the original sample enabling mapping lipid abundance with comparable quality to the tissue.

### Spatial lipidomics of HG replica

**Fig. 4:**
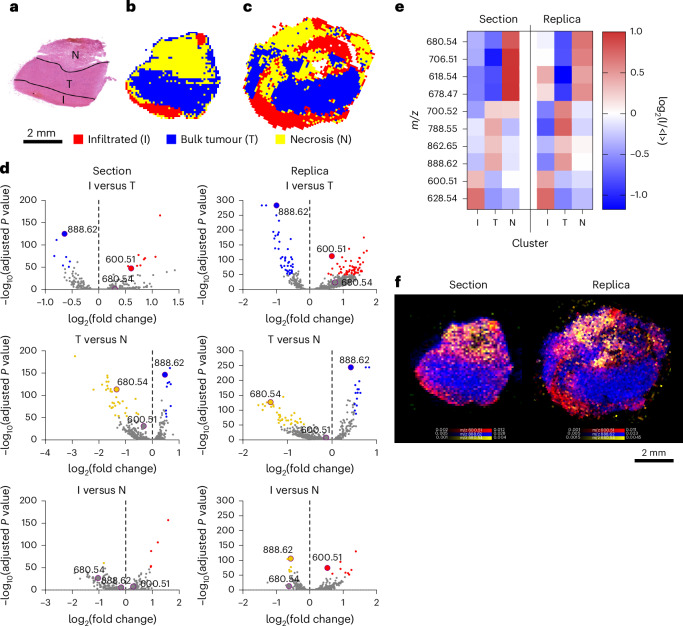
Spatial lipidomics of HG replica. **a**, A bright-field image of the haematoxylin and eosin (H&E)-stained section of HG biopsy, showing infiltrated tumour (I), bulk tumour (T) and necrosis (N). **b**,**c**, HCA maps of the top three clusters from DESI-MSI images of the section (**b**) and replica (**c**). **d**, Volcano plots showing the differential lipid abundance analysis across I, T and N. consistently identifying *m*/*z* 600.51 (Cer 36:1;O2) for I, *m*/*z* 888.62 (SHexCer 42:2;O2) for T and *m*/*z* 680.54 (Cer 43:0;O3) for N in section and replica. **e**, A heatmap of the ten most representative lipids defining I, T and N in both section and replica. **f**, An overlay map of TIC-normalized intensity for *m*/*z* 600.51 (Cer 36:1;O2, red), 888.62 (SHexCer 42:2;O2, blue) and 680.54 (Cer 43:0;O3, yellow). Source data

We then captured the molecular heterogeneity of a HG biopsy. Histopathological analysis identified three distinct regions of healthy brain infiltrated by tumour (I), bulk tumour (T) and necrosis (N) (Fig. 4a). HCA could distinguish these three regions and identify their lipid composition in the replica and the section (Fig. 4b–e). Mapping the distribution of the peak at *m*/*z* 600.51 (Cer 36:1;O2) characterized the infiltrated tumour, while *m*/*z* 888.62 (SHexCer 42:2;O2) distinguished the bulk tumour and *m*/*z* 680.54 (Cer 43:0;O3) preferentially associated with areas of necrosis (Fig. 4f).

### Machine learning inference of HG grade

To evaluate whether replicas could match the predictive power of original tissues, we collected DESI-MSI maps of molecular replicas (HG-r) and reference sections (HG-s) from 23 HG samples with known prognoses (Supplementary Table 2). Additional sections (HG 11, 12) and replicas (HG 6, 18) were collected for intrapatient and interreplica validations, for a total of 25 sections and 25 replicas.

**Fig. 5:**
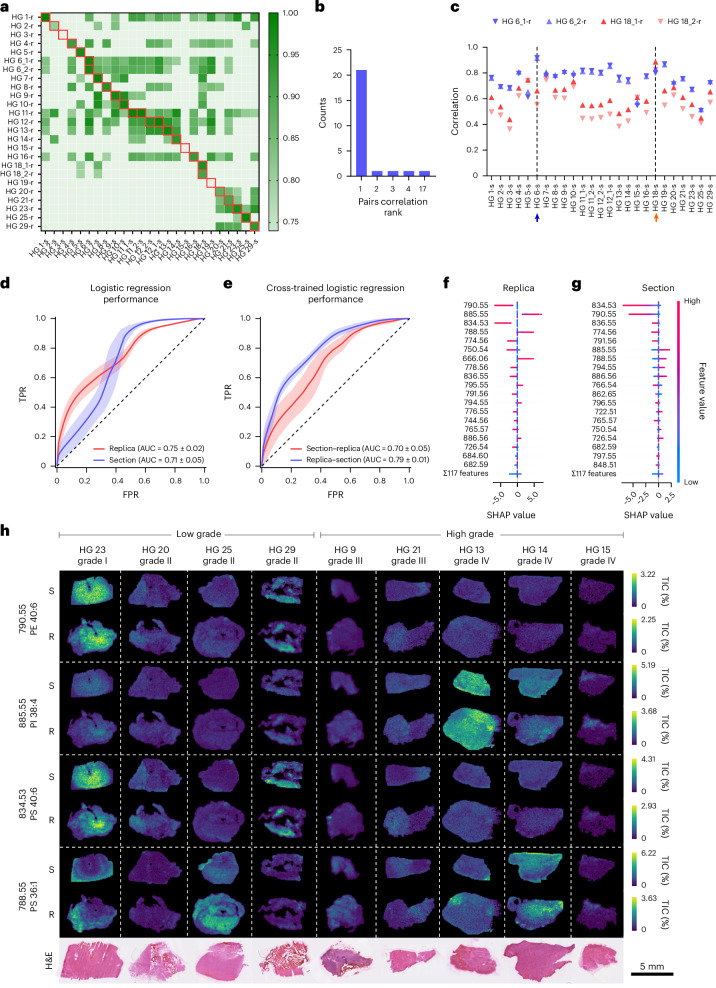
Machine learning inference of HG grade. **a**, A heatmap of correlation values between 25 tissue sections and 25 replicas from 23 biopsy samples. Samples are labelled HG plus a sequential number; multiple sections or replicas from the same biopsy include a trailing underscore and number. Sections are marked with ‘s’ and replicas with ‘r’. The red boxes indicate matched section–replica pairs. **b**, A count plot of correlation rank between matching tissue sections and molecular replicas. **c**, Pearson correlation analysis of the molecular replicas HG6_1-r, HG6_2-r, HG18_1-r and HG18_2-r, with all 25 sections. The arrows mark the reference HG6-s (blue) and HG18-s (orange) section. **d**, Receiver operator characteristics for the logistic regression classifier applied to the HG dataset. Data are mean ± s.d. from 100 random-seed classifications. **e**, Receiver operator characteristics for logistic regression cross-inference: model trained on sections, tested on replicas and vice versa. Data are mean ± s.d. from 100 random-seed classifications. TPR, true positive rate; FPR, false positive rate. **f**,**g**, SHAP value distributions of top-ranked features in the replica (**f**) and the section model (**g**). Positive SHAP values indicate contribution to high-grade predictions. **h**, TIC-normalized ion maps of selected replicas (R) and tissue sections (S) showing the top four peaks from **f**, alongside H&E-stained morphology. Source data

We observed a high correlation between replicas and their reference tissue section, indicating a preserved molecular profile (Fig. 5a). The correlation with the reference section ranked first with a probability of 0.85, and ranked in the top five with a probability of 0.96, well above the random expectations of 0.04 and 0.2, respectively (Fig. 5b). Replicas from the same section preserved a common molecular signature, showing higher correlation with their reference section than with any other (HG 6, 18) (Fig. 5c).

We next assessed disease grade prediction using sections and replicas comparing four classification models: logistic regression, decision trees, XGBoost^27^ and LightGBM^28^. All models performed better than a random model for both sections and replicas (Fig. 5d and Supplementary Fig. 10), with disease grade assignments showing a high degree of concordance. Logistic regression demonstrated superior performance (Fig. 5d and Supplementary Fig. 10) and thus was considered for further analysis. The classification performance was comparable for sections and replicas with a mean (±s.d.) area under the curve (AUC) for the receiver operating characteristic (ROC) of 0.71 ± 0.05 and 0.75 ± 0.02, respectively (Fig. 5d and Supplementary Table 3). Bootstrapping statistical validation confirmed that the observed classification performance was not due to chance (Supplementary Fig. 11). The best-performing models agreed on 25 out of the 27 section–replica pairs, further indicating that the models captured similar discriminative information from the spectral data (Supplementary Table 2). Cross-model prediction analysis applying the models trained on sections to predict the replicas achieved an AUC of 0.79 ± 0.01, while models trained on replicas to predict the sections achieved an AUC of 0.70 ± 0.05, further supporting the similarity in their molecular profiles (Fig. 5e and Supplementary Table 3). Shapley additive explanations (SHAP)^29^ showed that section and replica shared three of the top five most predictive peaks, while nine out of the top ten displayed the same correlation with lesion grade (Fig. 5f,g). Among the top four replica peaks, two (*m*/*z* 790.55 (PE 40:6) and *m*/*z* 834.53 (PS 40:6)) correlated with low-grade tumours and two (*m*/*z* 885.55 (phosphatidylinositol (PI) 38:4) and *m*/*z* 788.55 (PS 36:1)) with high grade. Their intensity maps across representative tumours aligned with expected tumour grades (Fig. 5h). This analysis also captured the spatial heterogeneity of tumours, identifying the lower section of HG 25—histologically infiltrated healthy brain—as low grade, enriched in the *m*/*z* values of 790.55 and 834.53, and the edges of HG 23 as high grade, enriched in *m*/*z* 788.55 and depleted in low-grade markers. These results established that the molecular replica generated using nanoneedles can replace a tissue section for disease state classification.

### Molecular replicas of living tissues

**Fig. 6:**
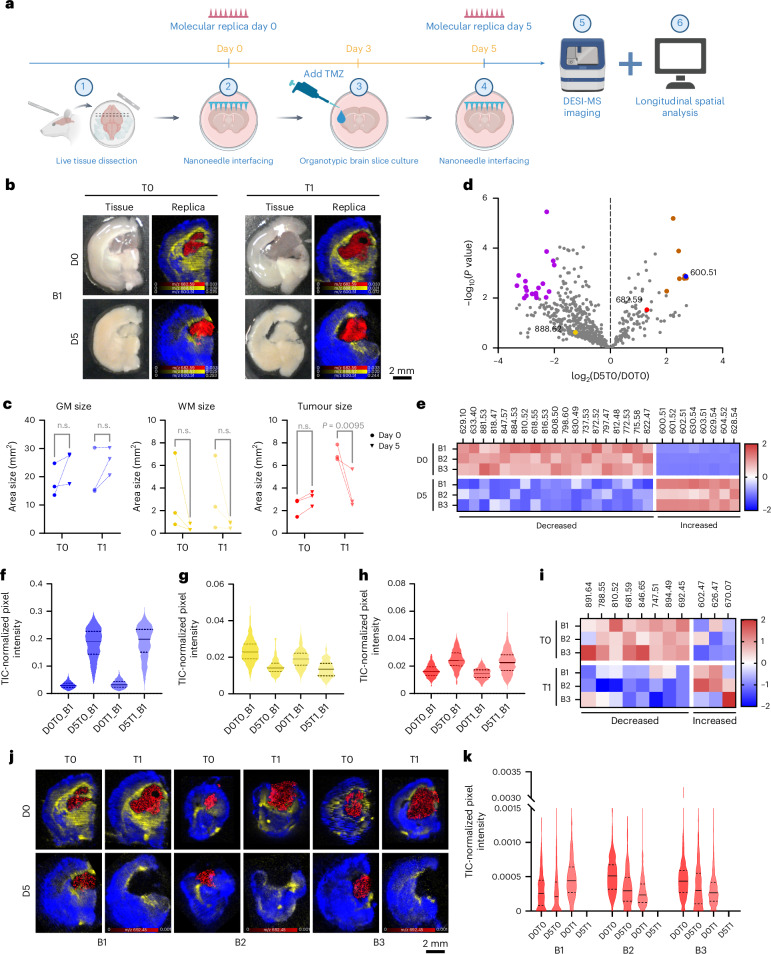
Spatiotemporal lipidomics of molecular replicas. **a**, A schematic of the longitudinal analysis workflow. Created with BioRender.com. **b**, Photographs (left) of brain 1 (B1) showing treated (T1) and untreated (T0) tissue slices at day 0 (D0) and day 5 (D5), with corresponding DESI-MSI maps (right) of grey matter (*m*/*z* 600.51, blue), white matter (*m*/*z* 888.62, yellow) and tumour (*m*/*z* 682.59, red) markers from molecular replicas of the photographed acute tissue slice. **c**, Quantification of grey matter, white matter and tumour areas from DESI-MSI molecular replicas data across time and treatment in three brain slices. Statistics: ordinary two-way ANOVA with post-hoc Šidák multiple-comparisons test. n.s., not significant. **d**, A volcano plot of lipid changes over time in untreated brains, highlighting markers for white matter (yellow), grey matter (blue) and tumour (red) and significantly increased (brown) or decreased (purple) species. *N* = 3 brain slices. **e**, A heatmap of the differentially abundant species (orange and purple) shown in **d**. **f**–**h**, Violin plots of grey matter (**f**), white matter (**g**) and tumour (**h**) marker abundance in brain 1 across time and treatment. Data: solid line: median; dashed lines: upper and lower quartiles. **i**, A heatmap of *z* scores for treatment-dependent significant lipids. **j**, DESI-MSI maps of treated and untreated molecular replicas at day 0 and day 5 for the lipid at *m*/*z* 692.45 (PS 29:0, red) in the tumour alongside grey matter (*m*/*z* 600.51, Cer 36:1;O2, blue) and white matter (*m*/*z* 888.62, SHexCer 42:2;O2, yellow) markers. **k**, Violin plots of *m*/*z* 692.45 (PS 29:0) abundance. Data: solid line: median; dashed lines: upper and lower quartiles. Source data

The non-destructive molecular collection enabled by nanoneedles offers a unique opportunity to sample living tissue longitudinally, allowing one to track its spatiotemporal lipidomic evolution. We tested this ability by monitoring the response of live glioma-bearing tissue slices to temozolomide (TMZ) treatment. We collected longitudinal molecular replicas on day 0 (D0T1, treated; D0T0, control) and day 5 (D5T1, treated; D5T0, control) while exposing the samples to TMZ or dimethylsulfoxide from day 3 (Fig. 6a).

**Extended Data Fig. 2:**
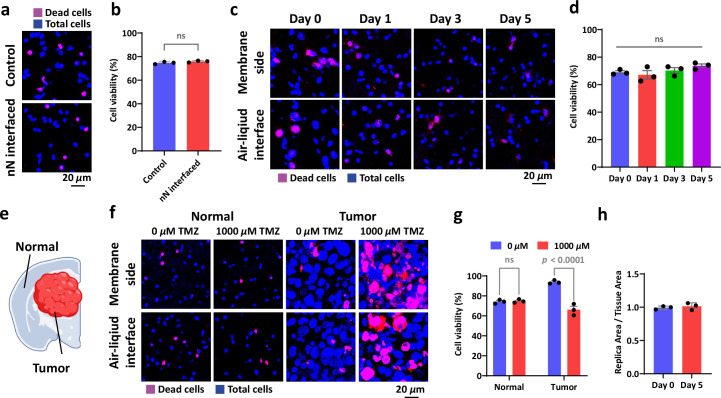
Molecular replicas of living tissues. (**a**) Fluorescence microscopy images of brain tissue slices showing total cells (blue) and dead cells (purple) after nanoneedle imprinting, compared with an untreated control. (**b**) Quantification of cell viability from (**a**). Data: mean with s.e.m. (N = 3 independent biological replicates). Statistics: two-tailed unpaired t-test. (**c**) Fluorescence microscopy images of total cells (blue) and dead cells (purple) in brain tissue slices over five days following first nanoneedle imprinting. (**d**) Quantification of cell viability from (**c**). Data: mean with s.e.m. (N = 3 independent biological replicates). Statistics: ordinary two-way ANOVA with post hoc Tukey’s multiple comparisons test. (**e**) Schematic indicating tumour and normal regions analysed in (**f**,**g**). Created in BioRender. Chiappini, C. (2025) https://BioRender.com/t7ha8ef. (**f**) Fluorescence microscopy images of normal and tumour regions showing total cells (blue) and dead cells (purple) after three days of culture with 1000 μM temozolomide or DMSO control. (**g**) Quantification of cell viability from (**f**). Data: mean with s.e.m. (N = 3 independent biological replicates). Statistics: ordinary two-way ANOVA with post hoc Šidák multiple comparisons test. (**h**) Quantification of the relative surface area of a molecular replica to its tissue sample on day 0 and day 5. Data: mean with s.e.m. (N = 3 independent biological replicates). Source data

We first assessed the feasibility of generating molecular replicas from living tissue with minimal perturbation. Nanoneedle imprinting did not impact brain slice viability immediately (Extended Data Fig. 2a,b) or over the 5 days of culture (Extended Data Fig. 2c,d), while the TMZ treatment selectively reduced the viability of cells in the tumour region by day 5 (Extended Data Fig. 2e–g). The day 0 and day 5 replicas captured the entire tissue slice area (Extended Data Fig. 2h).

### Spatiotemporal lipidomics of molecular replicas

We used these longitudinal molecular replicas to study the spatiotemporal variations in the lipidomic profile in response to TMZ treatment. The DESI imaging of the molecular replicas at both timepoints showed well-defined brain regions (Fig. 6b and Supplementary Fig. 12a,c). Tumour, grey matter and white matter were distinguished by the same lipids identified for the frozen sections (Figs. 3h and 6b). The morphology of these regions aligned well with the expected structure of the tumour-bearing brain and closely matched the brain structure observed in the slices, which evolved over the 5 days in culture. Over time, and regardless of treatment, the size of the grey matter slightly increased and the white matter decreased, consistent with the expected axonal degeneration in cultured brain slices^30,31^ (Fig. 6c). By contrast, the tumour size slightly increased in untreated samples while decreasing significantly in response to TMZ treatment, supporting the observation that the treatment selectively targeted tumour cells (Fig. 6c and Extended Data Fig. 2g).

We then assessed the temporal evolution of lipid composition by comparing lipid abundance between untreated samples at day 0 (D0T0) and day 5 (D5T0) across the three brains. Among the 629 commonly identified lipids on day 0, 21% increased in abundance over time, while 79% decreased with 31 of them becoming undetectable (Fig. 6d). Among these, 27 lipids showed significant changes, with 8 increasing and 19 decreasing in abundance (Fig. 6e). The white matter and tumour markers did not vary significantly, while the grey matter marker abundance increased over time (Fig. 6f–h and Supplementary Fig. 12b,d). The data indicate an overall decrease in the abundance and diversity of species as a consequence of culturing the brain slices. This analysis captures brain tissue remodelling during culturing and reveals changes in lipid composition and spatial distribution within individual slices, insights made possible through longitudinal sampling enabled by nanoneedles.

We then analysed the effect of treatment specifically within the tumour. Overall, the relative abundance of 42% of lipids increased with treatment, while 58% decreased. Statistical analysis identified 11 significant lipids (Fig. 6i), with 8 decreasing and 3 increasing in abundance with TMZ treatment. The most significant, *m*/*z* 692.45 (PS 29:0), selectively disappeared in treated tumours across all samples (Fig. 6j,k). Other lipid species showed distinct, treatment-dependent abundance patterns, highlighting the power of longitudinal molecular replica analysis to detect lipid-specific and specimen-specific treatment responses (Supplementary Fig. 13). Phosphatidylserines (PS29:0, PS 42:8, PS O-41:0, PS 38:4, PS 36:1) and phosphatidylinositol (PI O-39:1) featured prominently among the species decreasing in response to TMZ (Supplementary Table 1). Phosphatidylserine upregulation is a central feature of glioblastoma multiforme^32^ that is modulated by TMZ treatment^33^, including through ferroptosis^34–36^. The PI3K–AKT is a ubiquitously upregulated pathway in glioblastoma^37^, which is leveraged by TMZ^38^.

These data show that TMZ treatment alters tumour lipid composition and distribution, notably by selectively reducing specific metabolites. Using longitudinal data from molecular replicas, we characterized the response of the tumour and identified target lipids previously linked to glioma and TMZ. This analysis offers valuable insights into metabolic pathways affected by the treatment and potential biomarkers of therapeutic efficacy.

## Conclusions

We developed an imprinting workflow using porous silicon nanoneedles to generate reliable, repeatable molecular replicas of living tissue, enabling longitudinal lipid mapping for glioma characterization and classification. Interestingly, introducing a continuous porous layer reduced the quality of molecular replica, possibly due to dampening of the liquid-phase desorption–ionization process due to the absorbent nature of the porous matrix. A systematic investigation of the process would further improve replica quality.

These replicas well preserved the relative composition and spatial distribution of the original samples, allowing accurate mapping of brain regions and their lipid profiles. This approach enabled effective disease grade prediction, matching the performance of direct tissue analysis. Crucially, nanoneedles supported repeated, non-perturbing^12,39^ longitudinal sampling of glioma-bearing brain slices, revealing lipid-specific and sample-specific changes in tumour lipid profiles in response to chemotherapy.

By preserving lipid spatial distribution and enabling non-destructive, repeated sampling, nanoneedles offer a powerful platform for spatiotemporal, omics-level molecular diagnostics. Their integration into medical devices^40^, enables the analysis of accessible tissues, including intraoperative sampling during glioma surgery and longitudinal monitoring of mucosal surfaces. Looking ahead, advancing multi-omics integration, increasing spatial and temporal resolution, and accelerating analysis will be key to achieving comprehensive, real-time maps of tissue state evolution.

## Methods

### Porous silicon fabrication

Nanoneedles were fabricated according to our established protocols^12,41^. We photopatterned 0.6-μm dots with 2-μm spacing on p-type 0.001–0.02 Ω cm 100-mm Si wafers coated in 160-nm silicon-rich silicon nitride. The pattern was transferred into the silicon nitride layer via CHF_3_/O_2_ plasma reactive ion etching (RIE), followed by electroless Ag deposition (20 mM AgNO_3_ in 10% HF, 2 min) and subsequent metal-assisted chemical etching (H_2_O_2_:HF (10%v/v) 1:99, 7.5 min). SF_6_ plasma RIE shaped nanoneedles. Chips were diced (0.8 × 0.8 cm^2^) after fabrication.

Porous silicon layers were fabricated according to established protocols^40^. Electrochemical etching (HF 50%:EtOH = 1:3) in solution with current density of 34 mA cm^−2^ was applied for 60 s.

### Animal tissues

All animal experiments were performed under the authority of project licence PBE6EB195, Prof. Al Jamal Khuloud and personal licence I13B24EFC, Miss Nadia Rouatbi granted by the UK Home Office in compliance with the UK Home Office (1989) code of practice and European Union directive 2010/EU/63 for the housing and care of Animals used in Scientific Procedures and the UK Co-coordinating Committee con Cancer Research guidelines (1998). The murine brain and liver tissue used for interfacing strategy definition, molecule collection total quantification and DESI-MSI optimization were collected from wild-type adult CD1 mice, when the culling of the animal, to collect other organs, was already planned. The glioma murine model was established in a C57BL/6J^42^ background, injecting neural stem cells with concomitant Nf1, Pten, EGFR-vIII mutations (NPE)^43^. Mice ~6–8 weeks age were injected with 400,000 cells into the right hemisphere. Starting from the bregma, the stereotactic coordinates were as follows: 3 mm anterior, 1 mm lateral and 3 mm deep^44^. Roughly 2–3 weeks after the implantation of the tumours, animals were humanely euthanized, and the brains were collected. The maximum allowable tumour burden is approximately 10^10^ photons s^−1^. All the animals remained below this defined limit. Animals were not selected on the basis of sex, and sex was not recorded during experimentation.

### Human biopsies

Human tissue samples from patients with radiological evidence of a brain tumour were obtained from consented patients undergoing surgery at the Department of Clinical Neurosciences, NHS Lothian, Edinburgh. Tissue sample collection was approved by a local regional ethics committee (Lothian NRS Bioresource 20/ES/0061). Samples were snap frozen and stored until analysis at −80 °C. Tumour diagnosis was established from histological analysis.

### Generation of tissue replicas

The substrate of interest was placed in contact with the flat surface of a tissue specimen for less than 1 s for fresh frozen samples, or 10 s for live tissues, to allow the transfer of biomolecules to the substrate. After replica generation, the substrate was left to dry in air 1–2 min, then rapidly frozen on dry ice and finally transferred to storage at −80 °C.

### Electrospray ionization MS analysis of lipid extracts

Chloroform:methanol (2:1, 200 µl) was used to extract the lipids from replica and section. Seventy microlitres of liquid chromatography (LC)–MS grade water (Fisher Scientific, 10728098) was added, and the sample was vortexed for 10 s, then centrifuged (3 min, 845*g*, 4 °C). The organic layer was transferred to new tubes and evaporated under N_2_ flow; then a loading buffer was added: 4.625 mg of ammonium acetate (Sigma-Aldrich, 73594) in 210 μl of isopropanol (Fisher Scientific, 10684355):acetonitrile(Fisher Scientific, A955-1):water (2:1:1). Samples were centrifuged (15,871*g*, 3 min) to remove residues.

Electrospray ionization MS was perfomed in a Xevo G2-XS (Waters) using sensitivity mode, negative polarity, cone temperature 100 °C, voltage 40 V, CH_2_OH:H_2_O 0:1, with 0.1% v/v formic acid. Injection was performed with a flow rate of 5 μl min^−1^ (MassLynx 4.2). Spectra were acquired with 20,000 full width at half maximum (mass accuracy ≤1 ppm) mass resolution, over the range *m*/*z* 50–1,200.

### DESI-MSI

DESI-MSI was performed using a Xevo G2-XS Tof mass spectrometer with DESI ion source (Waters) controlled by MassLynx 4.2 and HD Imaging 1.4 softwares operated in sensitivity mode, negative polarity, cone temperature 150 °C, voltage 50 V, pixel size 50–150 µm and scan rate 100 µm s^−1^. Spectra were acquired with 20,000 full width at half maximum (mass accuracy ≤1 ppm) mass resolution, over the range *m*/*z* 50–1,200.

### DESI MS/MS for lipid identification

Tandem MS (MS/MS) was first performed on tissue sections using a standard DESI-MSI set-up with optimized collision energy for fragmentation. When lipid abundance was insufficient for identification by DESI MS/MS, the analysis was performed on brain lipid extracts by LC–MS/MS (Supplementary Information). To identify the MS/MS fragmented ions of target *m*/*z* values, spectra were analysed with LipostarMSI (Molecular Horizon)^45^. All MS/MS spectra were then cross-checked in lipid databases including MassBank (https://massbank.eu/), Lipidmaps (https://lipidmaps.org/), HMDB (https://hmdb.ca/) and SwissLipids (https://www.swisslipids.org/) and the scientific literature (Supplementary Table 1).

### DESI-MSI data preprocessing

We developed a preprocessing pipeline to create a common and meaningful representation of the lipidomic signature across tissue sections and replicas. The pipeline consisted of tumour segmentation and spectra alignement by lockmass using the prominent peak at *m*/*z* 885.5498 followed by total ion count (TIC) normalization. For the analysis in Fig. 2 and Extended Data Fig. 1 where unbiased feature detection was essential to compare across conditions and replica substrates, we generated the common representation by quantization of raw spectra followed by correction (see below). For the analysis in Figs. 3–6, we generated the common representation using the peak identification algorithm of HD Imaging (HDI, Waters) to ensure the biological relevance of the detected features.

Normalization and quantization are used to adjust the individual mass spectra in order to remove spectra-to-spectra variability within a given section or replica, generating a common representation with reduced dimensionality that is comparable across all sections and replicas. Each spectrum was normalized using TIC normalization^46,47^. In this study, we used equal width binning with bin widths of half the *m*/*z* resolution. All *m*/*z* values and the corresponding intensity values within the bin are represented by the bin centre *m*/*z* value and the intensity sum. We assume that the peaks do not slide from one bin to the other during the acquisition, causing errors in peak alignment. This is a reasonable assumption as the bin width is significantly smaller than the mass resolution of the data collected. However, the *m*/*z* values corresponding to ions can be identified more accurately by reducing the bin size. Focusing on the lipid range of 600–900 *m*/z, this process leads to a spectrum representation of size 24,000 dimensions.

Tissue segmentation is the process of separating the tissue spectra from background (non-tissue) spectra. Due to the high dimensionality of mass spectrometric data, we created a single-channel representative image using the accumulation of the selected representative channels *m*/*z* values that are associated with grey matter, white matter and tumour, respectively^48^ (TRIANB—*m/z* 794.55, 834.53, 886.60; LIVER (STD)—*m/z* 634.40, 794.55, 886.60; LIVER (OPT)—*m*/*z* 794.55, 834.53, 886.60; CHIP TYPE (flat_porous_substrate)—*m/z* 861.89, 848.89, 862.85; CHIP TYPE (porous_nNs_with_porous_substrate)—*m/z* 600.51, 768.53, 885.55; CHIP TYPE (porous_nNs)—*m/z* 794.55, 834.53, 886.60; CHIP TYPE (solid_nNs)—*m/z* 627.53, 834.53, 886.60; tissue section—*m*/*z* 794.55, 834.53, 886.60). Using the average threshold, we segmented the representative image to use as a mask for the separation of tissue and background spectra. For better segmentation and removal of salt and pepper noise, we used median smoothing with a filter size of 3.

Spectra correction according to the background (background correction) is the process of removing background signals that do not represent the actual tumour signal. Given a spectrum in an image, we applied *z*-score normalization using the mean and standard deviation (s.d.) of background spectra of the image. This ensures that the background signal has a mean of 0 and s.d. of 1.

### Section–replica disease state classification

Classification of tissue sections and replicas into high- and low-grade gliomas was performed using machine learning models from Scikit-learn^49^ applied to single-spectrum data within *m*/*z* 600–900 (lipids). This single-spectrum classification approach was selected to accommodate the limited cohort size and the assumption that each biopsy exhibited a homogeneous grade. Models were trained separately for sections and replicas, utilizing four machine learning algorithms: logistic regression, decision trees, XGBoost^27^ and LightGBM^28^.

To ensure robustness and prevent data contamination, we used a leave-one-batch-and-patient-out validation strategy (Supplementary Table 4). Specifically, models were trained on data excluding all spectra from the same patient and imaging session as the test set. This cross-validation approach mitigated potential confounding effects of batch variability and patient-specific biases. Hyperparameter tuning for each model was conducted using the Optuna^50^ framework, optimizing for the ROC AUC.

To evaluate the variability and stability of model performance, the classification process was repeated across 100 different random seeds. The primary evaluation metric was the ROC AUC, calculated independently for sections and replicas. In addition, cross-prediction analyses were performed, with models trained on sections tested on replicas, and vice versa, to assess the molecular similarity between the two.

For the best-performing algorithm, logistic regression, further analysis and interpretation was done. Permutation testing validated the significance of classification performance, comparing median AUC values against those obtained from randomized labels. Results were complemented by an analysis of feature importance using SHAP^29^ to identify significant spectral peaks contributing to classification.

### Longitudinal analysis

The data were analysed using custom scripts, starting from the HDI common lipid representation. Longitudinal changes in untreated samples were assessed using analysis of variance (ANOVA) on a univariate linear regression model, comparing lipid abundance between D0T0 and D5T0. Changes were considered significant if *P* < 0.01 and $|{{\mathrm{log}}}_{2}(\frac{{\mathrm{D}}5{\mathrm{T}}0}{{\mathrm{D}}0{\mathrm{T}}0})| > 2$ with each brain treated as an individual sample. To study the effect of treatement on the tumour for each species, we defined the metric $\Delta T=\Delta {\mathrm{T}}1-\Delta {\mathrm{T}}0$ where $\Delta {\mathrm{T}}0=\left[{\mathrm{D}}5{\mathrm{T}}0\right]-[{\mathrm{D}}0{\mathrm{T}}0]$ and $\Delta {\mathrm{T}}1=\left[{\mathrm{D}}5{\mathrm{T}}1\right]-\left[{\mathrm{D}}0{\mathrm{T}}1\right]$ changes in species abundance over time for untreated and treated samples, respectively. Abundances were calculated as the average across all pixels classified as tumour. Each tissue slice was considered a separate sample. A negative $\Delta T$ indicated a net decrease in species abundance due to treatment, while a positive $\Delta T$ indicated a net increase. Treatment-related changes were identified by performing ANOVA on a linear regression model with time and treatment as factors. Changes were deemed significant if *P* < 0.01 and $\left|z\left(\Delta T\;\right)\right| > 0.5$ where $z\left(\Delta T\;\right)$ is the *z* score of $\Delta T$. Model robustness was tested by including cross-factor interactions and sample pairing, which showed high concordance in identifying significant species.

### Data analysis

No statistical methods were used to predetermine sample sizes, but our sample sizes are similar to those reported in previous publications^4,13,51^. Data distribution was assumed to be normal, but this was not formally tested. Samples were randomly selected for assignment to the experimental groups to ensure unbiased distribution across conditions. Experimental conditions and stimulus presentation were also organized without any systematic bias to ensure that each sample had an equal chance of being exposed to any given condition. Data collection and analysis were not performed blind to the conditions of the experiments. No animal or data point was excluded from the analysis. Statistical analysis and graphical data visualization was done using GraphPad Prism 9 and 10 (GraphPad). For volcano plots, differences between two groups were calculated by Student’s *t*-test. Peaks with log_10_(*P* value) <50 and abs(log_2_(fold change)) >1 or >0.5 informed the selection of peaks relevant for discrimination of tissues and confirmed by visual inspection.

### Reporting summary

Further information on research design is available in the Nature Portfolio Reporting Summary linked to this article.

## Online content

Any methods, additional references, Nature Portfolio reporting summaries, source data, extended data, supplementary information, acknowledgements, peer review information; details of author contributions and competing interests; and statements of data and code availability are available at 10.1038/s41565-025-01955-8.

## Supplementary information

Supplementary Information Supplementary materials and methods, Figs. 1–13 and Tables 1–4.

Reporting Summary

## Source data

Source Data Fig. 2 Statistical source data.

Source Data Fig. 3 Statistical source data.

Source Data Fig. 4 Statistical source data.

Source Data Fig. 5 Statistical source data.

Source Data Fig. 6 Statistical source data.

Source Data Extended Data Fig. 1 Statistical source data.

Source Data Extended Data Fig. 2 Statistical source data.

## Data Availability

All data presented in this Article, including all MS datasets, are available via the KORDS repository at 10.18742/c.7711667. Source data are provided with this paper.

## Code availability

The source code developed for the data analysis is available via GitHub at https://github.com/ChiappiniLab/Nanoneedle-Lipidomics and https://github.com/zaritskylab/Nanoneedle-Lipidomics.
