# Supplementary material for: Nanoneedles enable spatiotemporal lipidomics of living tissues
Source: Nat Nanotechnol. 2025 Jun 16;20(9):1262–72. doi: 10.1038/s41565-025-01955-8 (PMC12443637; doi:10.1038/s41565-025-01955-8)
Supplement: Supplementary file 1 — Supplementary materials and methods, Figs. 1–13 and Tables 1–4. [file 41565_2025_1955_MOESM1_ESM.pdf]

nature nanotechnology

Supplementary information

https://doi.org/10.1038/s41565-025-01955-8

Nanoneedles enable spatiotemporal lipidomics of living tissues

In the format provided by the authors and unedited

Nanoneedles Enable Spatiotemporal Lipidomics for Glioma Profiling

Chenlei Gu, Davide Alessandro Martella, Leor Ariel Rose, Nadia Rouatbi, Cong Wang, Alaa Zam, Valeria Caprettini, Magnus Jensen, Shiyue Liu, Cathleen Hagemann, Siham Memdouh, Andrea Serio, Vincenzo Abbate, Khuloud T. Al-Jamal, Maddy Parsons, Mads S. Bergholt, Paul Brennan, Assaf Zaritsky, Ciro Chiappini

Table of Contents

Supplementary Materials and Methods...2
Supplementary Figures and Tables 7
References...27

Supplementary Materials and Methods

Cryosectioning of tissue

The tissue sections were collected with a Bright OTF5000 cryotome. The chamber and specimen temperature were set at -20 °C. The sections were collected onto glass slides, superfrost® plus slides (fisher scientific 12625336).

Vibratome sectioning of live tissue

Mice were humanely sacrificed and decapitated under sterile conditions. The brains were immediately dissected and transferred into prechilled RPMI 1640 medium (Gibco). The live brains were transported on ice and embedded in 4% low melting point agarose (R0801, Thermo Scientific) prepared in 1x DPBS (D8537, Sigma-Aldrich). Live brain sections (400 µm thickness) were collected with a vibratome (Leica VT1000 S) with the ice bath filled before sectioning.

SEM imaging

SEM images of the substrates and molecular replicas were acquired with the in-lens detector using a Karl Zeiss XB1540 SEM at 10 kV, after 10 nm Au sputter coating to avoid charging effects.

Histology of tissue sections

Tissue sections were processed by H&E using an automated LeicaBOND tissue processor. After completion of the mounting at 60°C overnight, the stained sections were imaged by the slide scanner Hamamatsu Nanozoomer. The software NDP view 2 (Hamamatsu) was used for visualization and annotations of H&E stained slides.

Lipid staining of tissue replicas on flat and nanoneedles

Lipophilic carbocyanine dye Neuro-DiO (Biotium, 30021) was used to stain the lipid of tissue replicas according to the manufacturer protocol. In brief, the tissue replicas were first fixed using paraformaldehyde (4% in PBS) for 10 minutes at room temperature. After PBS (1x) washing 3 times, enough freshly prepared Neuro-DiO (in 1x PBS) staining solution was added to cover the nanoneedles, then incubated 30 minutes in the dark at room temperature, and finally washed 3 times with PBS. Tile scan images were acquired by Leica DMi8 inverted microscope (Leica Microsystems GmbH) with 20x 0.4 NA air objective.

Extraction and total quantification of biomolecules

RNA. All surfaces were wiped with RNase AWAY™ (Thermo Scientific™ 10666421). Biomolecules were extracted with 300 µL TRIzol. To assist the elution of biomolecules from 1 cm^2 nNs and glass chips, ceramic beads (MP Biomedicals, Lysing matrix D, 2 mL tube, 6913050) with 20 s, 4 m/s tissue homogenisation (MP Biomedicals FastPrep-24) were used. The solution was centrifuged at 16,000 x g for 1 min and the supernatant was collected, to remove the nNs and glass residue. Direct-zol™ RNA MiniPrep Plus (Zymo research, R2070S) was employed for RNA extraction. An equal volume of ethanol 100% was added to the sample before transferring it to a Zymo-Spin™ IIICG Column (Zymo research, C1006). The sample was centrifuged at 16,000 x g for 30 s, and the flowthrough underwent to protein extraction. The column was transferred to another RNase free tube and underwent 2 pre-washes and 1 wash step using the buffers provided with the columns, as per manufacturer instruction. Finally,

the RNA was collected in RNase free water. Qubit™ RNA HS assay (Thermo Fisher Scientific, Q32852) was employed for total RNA detection, following the user manual from the manufacturer, using a Qubit 3.0 fluorometer (Invitrogen™ Q33216).

Proteins. The flowthrough from the RNA extraction in the column was incubated for 30 min on ice, after adding 4 volumes of cold acetone (-20 °C). After centrifugation at 20,000 x g for 10 min, the protein pellet underwent ethanol wash and centrifugation at 20,000 x g for 1 min. The protein pellet was air dried for 10 min at room temperature, then resuspended in 100 µL protein storage buffer: 4 M urea (Sigma-Aldrich, U5128), 1 % SDS (Sigma-Aldrich, L3771). Proteins were kept at -80 °C until quantification. The detergent was removed from the samples in storage buffer by loading 100 µL of sample in pre-dispensed HiPPR™ spin columns (Thermo Scientific™, 88305), then following the user manual from the manufacturer. Desalting was performed by loading 100 µL of sample in Pierce™ Protein Concentrators PES, 3K MWCO (Thermo Scientific™, 88512), following the user manual from the manufacturer. The protein concentrator allowed to change the storage buffer to 100 µL of 0.1 M sodium borate (Sigma-Aldrich, HT1002), pH 9.3, for total quantification by CBQCA protein quantitation kit (Invitrogen™, C6667). A standard curve using bovine serum albumin (Sigma, A9647) was built following the manufacturer instruction. Samples and standards were processed according to the user manual from the manufacturer. Fluorescence was measured using the CLARIOstar® Plus microplate reader (BGM Labtech), with excitation waveband 465 ± 15 nm and emission waveband 550 ± 20 nm.

Lipids. Biomolecules were extracted with 200 µL CH_2OH-H_2O mixture or $CHCl_3$. To assist the elution of biomolecules from nNs and glass chips ultrasonication or ceramic beads (MP Biomedicals, Lysing matrix D, 2 mL tube, 6913050) with 20 s, 4 m/s tissue homogenisation (MP Biomedicals FastPrep-24) were used. Samples were immediately placed on ice. The solution was centrifuged at 16,000 x g for 1 min and the supernatant was collected, to remove the nNs and glass residue. The ratio of $CHCl_3$-CH_2OH-H_2O was brought to 1:2:0.8 and the sample mixed, then $CHCl_3$ was added to obtain ratio 2:2:0.8, vortexed for 30 s and allow phase separation. The aqueous layer was removed. The organic phase was transferred into new tubes for quantification by Nile Red staining. A stock solution was prepared for Nile Red (NR) (9-diethylamino-5H-benzo[α]phenoxazine-5-one, $C_{20}H_{18}N_2O_2$, Sigma Aldrich 72485) in acetone 1:40 v/v. The samples were dried under N_2 flow to 1/10 of the original volume or until complete evaporation. A volume of 200 µL of water was added to the samples. Immediately after vortexing the samples, 2 µL of NR stock solution was added to the samples. The samples were then vigorously vortexed for 1 min to obtain a microemulsion. The samples were finally transferred to a well plate for analysis. Fluorescence was measured using CLARIOstar® Plus microplate reader, with excitation waveband 530 ± 15 nm and emission waveband 612 ± 100 nm.

DESI-MSI optimisation

The total lipid extract from porcine brain (Avanti® Polar Lipids, Inc. 131101C) was diluted in methanol to obtain concentrations between 8 and 320 ng/μL. For each concentration 10 μL were drop deposited onto the nNs and flat surface. The surfaces were dried in air. Three distinct samples were imaged for each concentration. The spectra were acquired by a Xevo® G2-XS Tof mass spectrometer equipped with DESI ion source (Waters™, Milford, MA, USA) controlled by MassLynx 4.2 software.

LC-MS/MS analysis

Lipids were extracted using a modified biphasic solvent system of cold methanol, MTBE, and water[1,2]. Mouse brain slices were mixed with 300 µL cold methanol, vortexed, and combined with 1 mL MTBE in a glass centrifuge tube. After sonication (VCX 130, 50% amplitude, 1 min) and 1-hour shaking at room temperature, 250 µL ultrapure water was added to induce phase separation, followed by 10 minutes of shaking. The mixture was centrifuged (2,000 x g, 10 min, 4 °C), and the upper organic phase was collected. A second extraction with 300 µL MTBE was performed, and both organic phases were pooled, concentrated to one-tenth volume under nitrogen, fully dried in a vacuum desiccator, and stored at –80 °C.

Untargeted lipidomics was performed with modifications[3], using a Thermo Scientific Q-Exactive™ mass spectrometer (Thermo Scientific, Germany) coupled with a Waters Acquity UPLC® (Waters Corp., USA). The HESI-II source used nitrogen with the following settings: sheath gas flow rate 60, auxiliary gas flow rate 25, spray voltage 3.0 kV, capillary temperature 300 °C, and auxiliary gas heater 370 °C. Full scans were first acquired in negative mode from m/z 80–1200 with a resolution of 35,000 FWHM and automatic gain control (AGC) target of 3×10^6. Parallel reaction monitoring (PRM) followed in negative mode (at 17,000 FWHM, AGC target of 2×10^5, isolation window of 1.2 m/z, and stepped normalised collision energy (NCE) was applied at 20, 30, and 45). Two targeted PRM methods included specific m/z within their inclusion lists: (1) 858.5290 and 891.6331 and (2) 692.4508, 747.5182 and 810.5290. Data were analyzed in Xcalibur™ (version 4.1, Thermo Scientific) and 5 ppm extraction window was set for the extracted ion chromatograms of the singly deprotonated ($[M - H]^-$) ions. The extracted lipids were reconstituted in methanol (200 µL) , vortexed (30 s), centrifuged (21,130 x g, 5 min), and the supernatant was stored at 4 °C for LC-MS/MS analysis. Chromatographic separation used the Acquity® UPLC BEH C18 reversed-phase column (particle size 1.7 µm, 2.1 X 50 mm; Waters, USA) at 65 °C, 0.6 mL/min flow, and 5-10 µL injection volume. Mobile phase A: 0.1% acetic acid in : acetonitrile:water (60:40) with 10 mM ammonium acetate; mobile phase B: 0.1% acetic acid in isopropanol:acetonitrile:water (90:10:0.1) with 10 mM ammonium acetate. The gradient started with 15% B, then to 30% over 1 min, 48% over 0.3 min, 76% over 3.5 min followed by 99% over 0.1 min and remained for 0.4 min before returning to the initial condition over 0.1 min. The column's equilibration time at the initial gradient condition followed over 1.9 min. The total run time: 7.30 min.

Section-Replica Correlation analysis

Correlation analysis for every section-replica pair was done by evaluating the Pearson correlation between their mean intensities spectra in the mass range *m/z* value of 600-900. This analysis produced a matrix of size nxm where n is the number of sections and m is the number of replicas. Bin (i,j) in this matrix indicates the correlation between section i and replica j. For each replica, we ranked all sections according to their correlation to quantitatively assess whether a replica is most similar to its matched section. To determine whether multiple replicas from the same section maintained their molecular signature, we measured the correlation between each of the replicas of the same tissue with their reference section versus all other sections.

Organotypic brain slice culture

Brain slices were cultured using the air-liquid interface method[4,5]. Specifically, 2 to 4 slices were transferred to one 0.4 μm Millicell membrane insert (PICM0RG50, Milipore) in a 6-well

plate using a micro spatula and paint brush. The culture medium was prepared with Neurobasal-A (Gibco), 2% B-27 Plus (Gibco), 2 mM GlutaMAX (Gibco) and 1x Penicillin-Streptomycin (Gibco). The membrane inserts were incubated in culture medium at least 10 minutes before the brain slices transfer. Slices were cultured in an incubator with 5% CO_2 at 37 °C since then. On day 3 half volume of the medium was changed with fresh ones either containing 2 mM temozolomide (T2577, Merck Life Science) prepared in dimethyl sulfoxide (DMSO, D2650, Sigma-Aldrich), or the equivalent DMSO concentration (less than 1%) as control. The treated brain slices were cultured for two more days in the incubator with 5% CO_2 at 37 °C.

Cell viability staining and quantification

Live brain slices were stained with the Fixable Viability Dye eFluor™ 780 (65-0865, Invitrogen, 1:1000 dilution in 1x DPBS) or Live-or-Dye NucFix™ Red Staining Kit (32010, Biotium, 1:500 dilution in 1x DPBS)) for dead cells according to the manufacture's protocols. For viability quantification at Day 0, the prepared slices were incubated with the fixable dye for 30 minutes on ice, washed with 1x DPBS three times, and then fixed in 4% paraformaldehyde at room temperature for at least 1 h. After three times with 1x DPBS, the fixed slices were incubated with Hoechst 33342 (Thermo Scientific, 1 μg/mL in 1x DPBS) for 30 minutes at room temperature. After three times wash in 1x DPBS, the stained brain slices were mounted onto a coverslip (Waldemar Knittel Glasbearbeitungs – GmbH) using the ProLong Gold Antifade Mountant (P36930, Invitrogen). For viability quantification at Day 1, 3 and 5, the cultured brain slices were gently rinsed in 1x DPBS three times to remove the culture medium before proceeding to the fixable dye staining. Confocal (Zeiss LSM 980) images were taken using the corresponding Ex/Em setup with a 20X, 0,8 NA objective. The cell viability was quantified using Fiji by 1 minus the dead-to-total cells ratio.

COMSOL finite element simulations

The finite element simulations were performed by using COMSOL Multiphysics® 5.4 and Heat Transfer module. Two geometries were defined: a rectangle 525 µm wide and 8 mm tall to simulate the nN chip and a rectangle 19.475 mm wide, 4 mm / 8 mm tall, to simulate a tissue specimen with smaller or larger contact area with the nN chip, respectively. A triangular 0.6 mm mesh was defined for both nN chip and tissue. The material properties were selected from the COMSOL library: silicon for the nN chip and water/ice for the tissue. The initial temperature of the nN chip was 25 °C, the initial temperature of the tissue was -20 °C. The side of the tissue further from the nN chip was kept constant at -20 °C, at all times during the simulation. An insulating layer was defined around all the other sides of the objects but the one where they were in contact. A time range of 1 s and step of 0.05 s were used for the simulation.

Principal Component Analysis

We used principal component analysis (PCA) to perform an internal validation of the clustering analysis, by assessing the DESI-MSI spectral variance between and within clusters. Mapping the distribution of principal component scores also provided further insight into the heterogeneity within the tumour cluster. The first principal component, PC 1 accounted for the 68 % of the variance, arising from the variation of the main spectral profile. The second and third principal components, PC 2 (6.8 %) and PC 3 (4.6 %) accounted for a large proportion of the variance within the dataset (Supplementary Figure S6a). The loading from PC 2 showed

positive peaks associated to white matter, in particular the sulfatide peak at m/z value of 888.62 (SHexCer 42:2;O2) and phosphatidylserine at *m/z* value of 788.55 (PS 36:1)[6]. The negative peaks were mainly associated to grey matter and tumour, in particular the phosphatidylserine peak at *m/z* value of 834.53 (PS 40:6, grey matter), and the phosphatidylinositol peak at *m/z* value of 885.55 (PI 38:4, grey matter and tumour)[6,7]. The loadings from PC 3 showed positive peaks associated to grey matter such as the *m/z* values of 600.51 (Cer 36:1;O2), and 834.53 (PS 40:6) and negative peaks associated to the tumour at such as the *m/z* values of 716.52 (PE 34:1), and 788.55 (PS 36:1). The score maps for PC 2 and PC 3 highlighted the association of the loading of individual components to the different tissues (Supplementary Figure S6b). The map of PC 2 scores distinguished white matter from grey matter and tumour. The map of PC 3 scores distinguished the tumour against grey matter and white matter.

Principal component analysis in combination with the assigned pixel identity from hierarchical cluster analysis provided further insight into the inherent spectral variability of tissue section and replica (Supplementary Figure S6c). For white and grey matter, the data from the replica showed a broadening of distribution and a shift towards null score. The tumour cluster instead remained more similarly distributed between section and replica. On both section and replica, PC 3 distinguished between tumour and grey matter, with the white matter in between. The grey matter distribution of PC 3-scores showed a bimodal distribution. The white matter cluster in the replica showed a shift towards the grey matter. In both section and replica, the tumour cluster showed a sharp distribution with a tail towards the other clusters. Spatially, these tails localised to the margin region between tumour and healthy tissue both in the section (Supplementary Figure S6d) and in the replica (Supplementary Fig S6e), suggesting an ability of PCA analysis to map the margin region of the tumour and the transitions between tissues.

The principal component analysis indicates that the information contained within the data from the replica broadly aligns with that of the original sample. Moreover, the intracluster variance in PCA loadings can map tumour margins and transitions between tissues in the replica as well as in the section.

Supplementary Figures and Tables

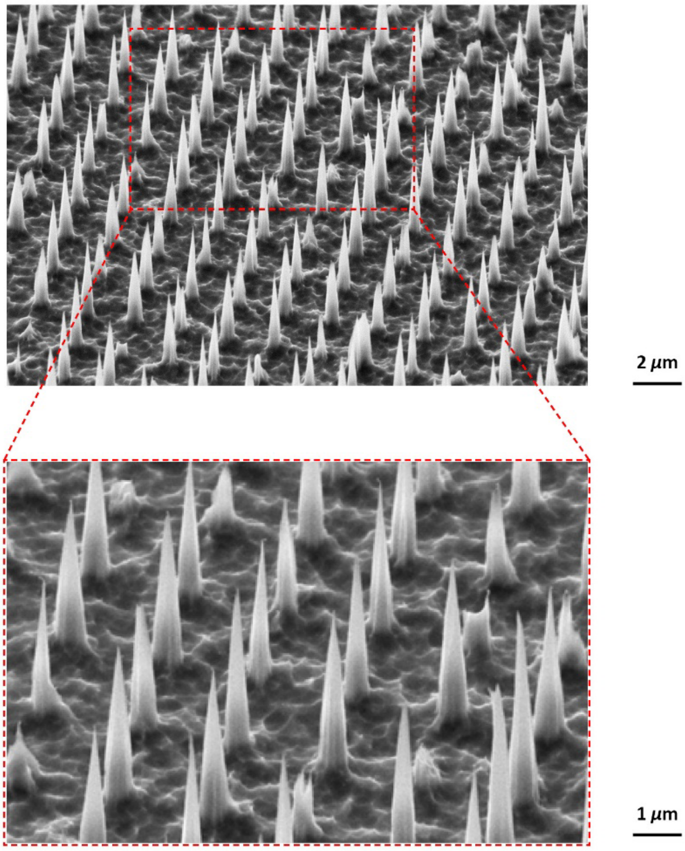

Figure S1: Scanning electron micrographs of the nanoneedles.

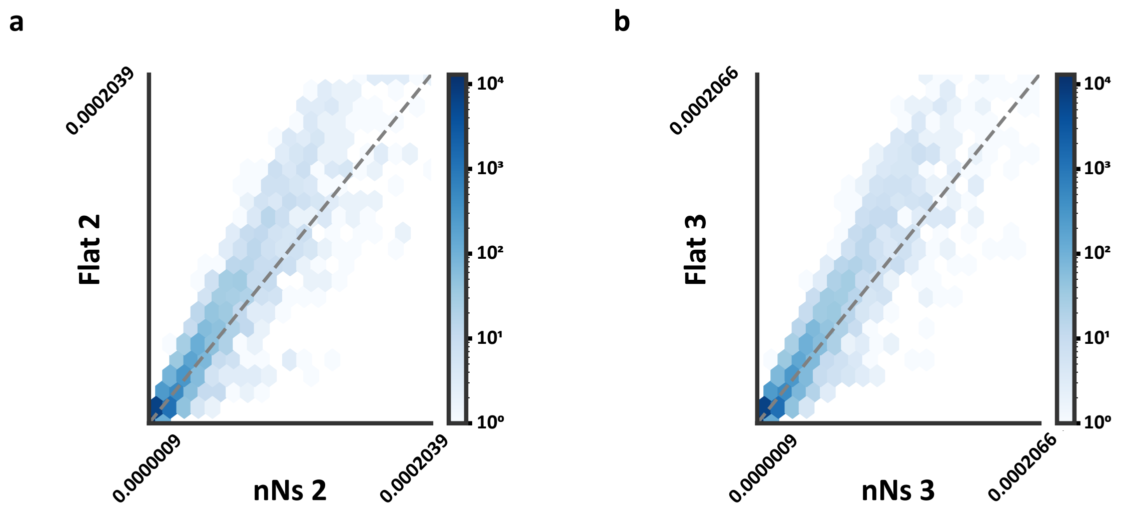

Figure S2: Correlation scatter plot comparing the relative intensity of lipids peaks between flat silicon and nanoneedles (nNs) for the second and third replicate.

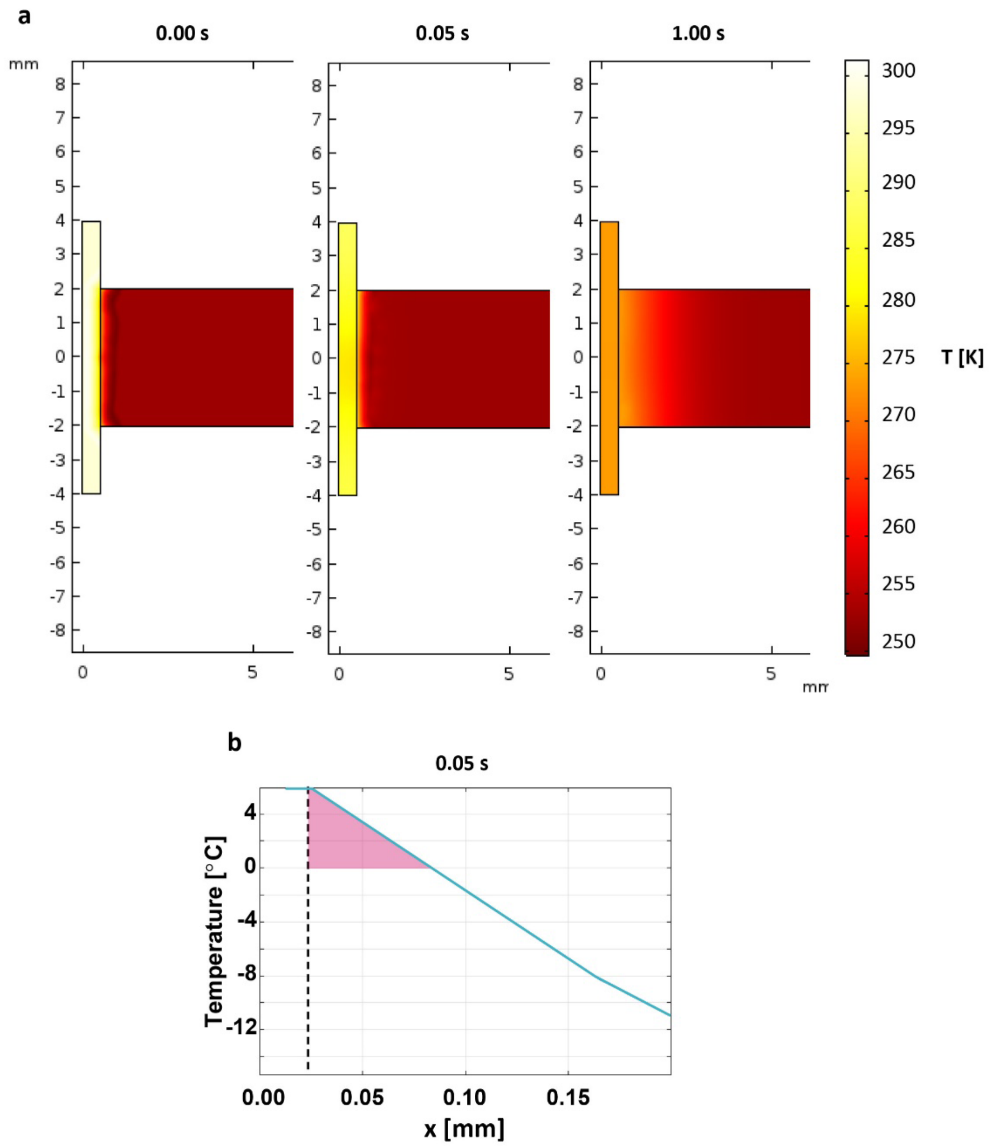

Figure S3: Computational modelling of tissue interfacing. (a) Cross-sectional snapshots of the simulation of heat transfer between chip and tissue. The chip is the tall and narrow element on the left while the tissue is the longer element on the right. From left to right the images show the distribution of the temperature at 0 s, 0.05 s and 1 s of the simulation. The chip was initially at 25 °C = 298.15 K, while the specimen was at -20 °C = 253.15 K. In the first 0.05 s, at the interface with the chip, the temperature of the specimen rose above 0 °C = 273.15 K. At 1 s the specimen re-froze onto the chip. (b) Plot of the temperature variation along a line drawn from the chip to the specimen, at 0.05 s. The dashed line marks the interface between chip and specimen. The area highlighted in pink shows the thawed region of ~60 µm within the tissue after 0.05 s.

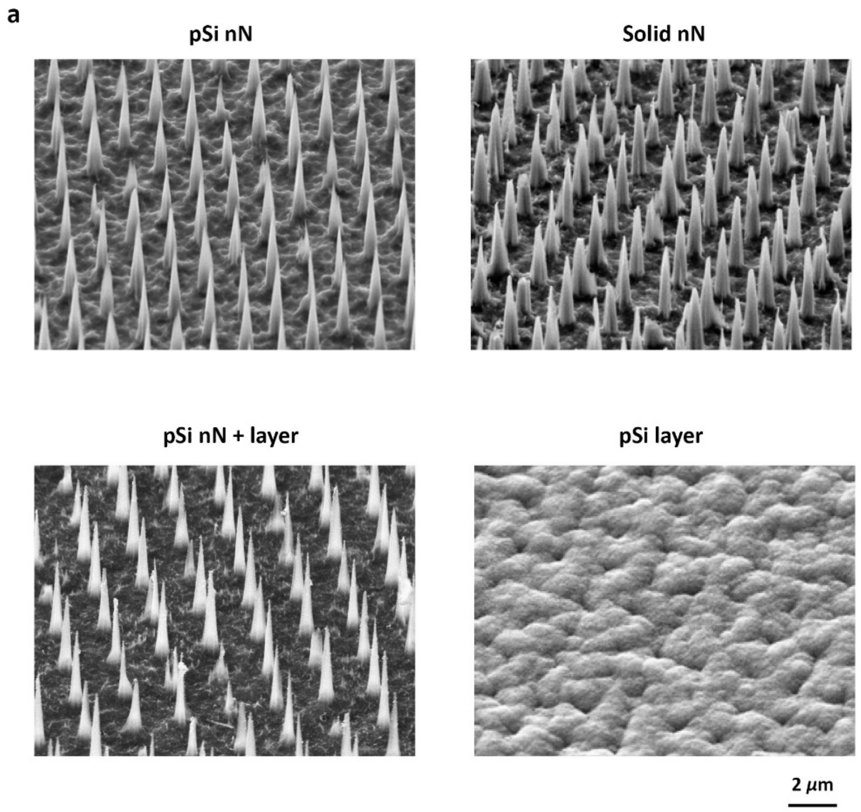

Figure S4: Scanning electron micrographs of the substrates used for assessing molecular replica quality: porous silicon nanoneedles (pSi nN), solid silicon nanoneedles (solid nN), porous silicon nanoneedles over a continuous porous silicon layer (pSi nN + layer) and a continuous porous silicon layer (pSi layer).

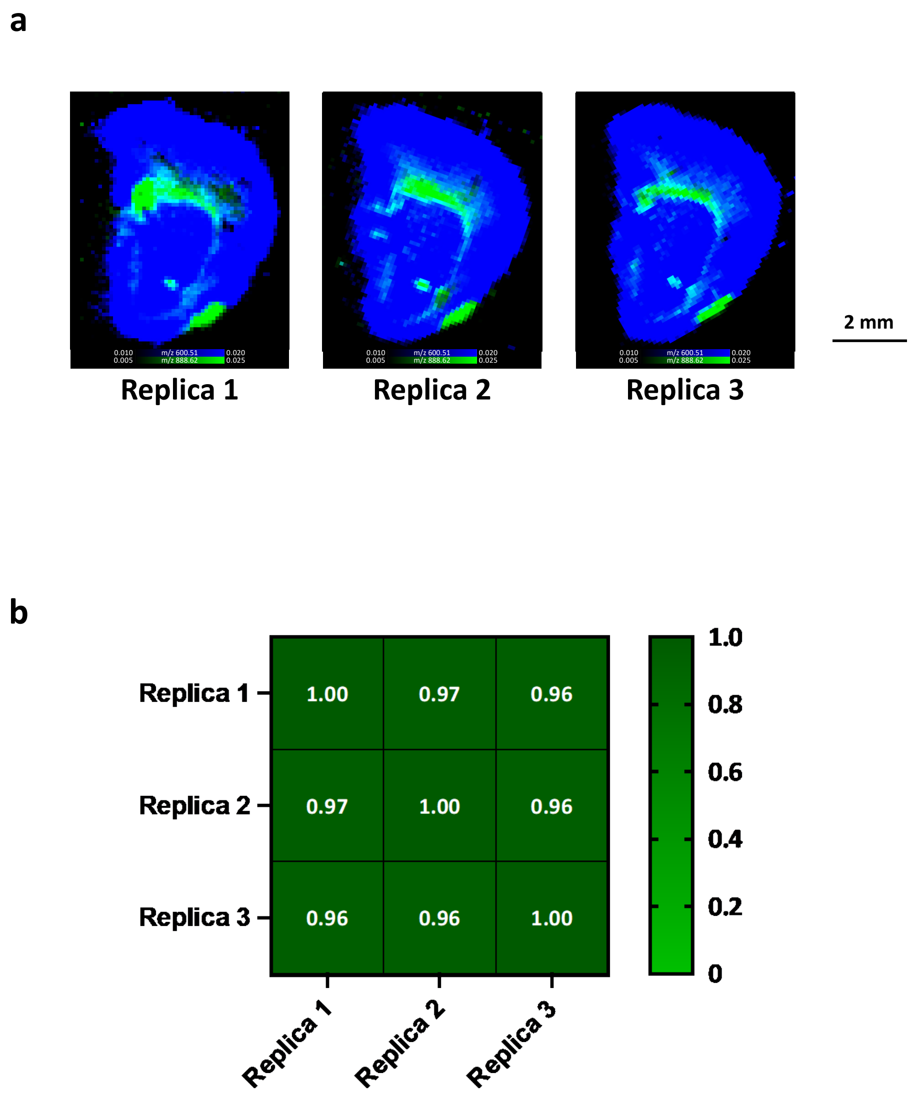

Figure S5: DESI imaging of three replicas from the same tissue, demonstrating (a) well-preserved, reproducible tissue morphology and (b) high inter-replica correlation.

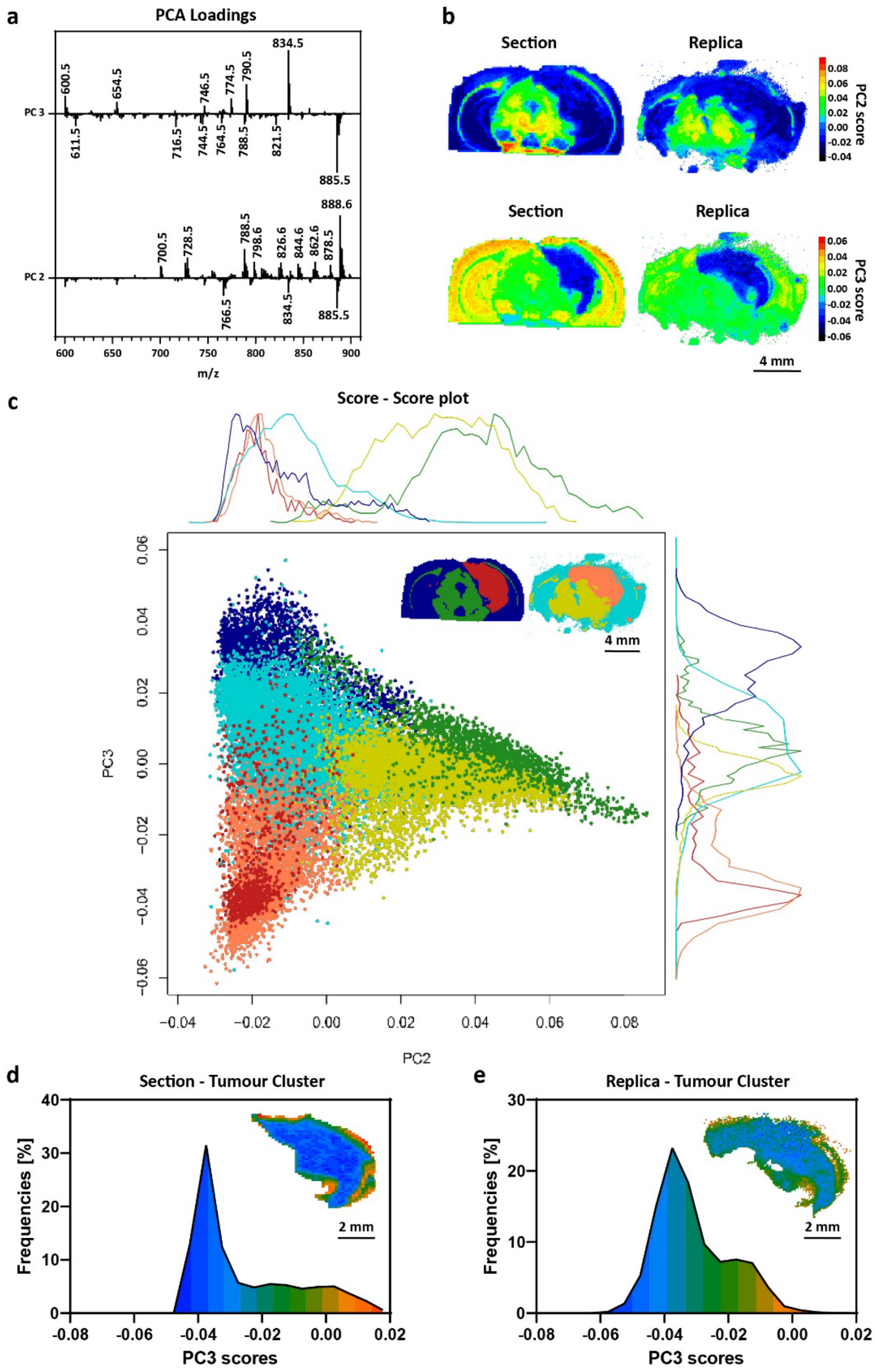

Figure S6: Principal Component Analysis. (a) Loadings of the principal components PC2 and PC3, obtained by principal component analysis (PCA) of DESI-MS images acquired from matching section and replica of tumour-bearing murine brain. (b) Maps of the scores from PCA for PC2 and PC3, showing that PC2 discriminates white matter vs the other 2 tissues, while PC3 discriminates healthy tissue vs tumour. (c) Score-score plot, showing the score value for every spectrum in section and replica. The colours were associated to the clusters as obtained by HCA and spatially distributed as shown in the inset cluster maps, to allow the observation of inter- and intra-cluster spectral variance. Along the axis, graphs representing spectra frequency distributions vs score of the principal component, for each cluster associated to grey matter/white matter/tumour and section/replica. (d) Graph representing the spectral frequency distribution of PC3 scores in the tumour-associated cluster in the section, with

colours showing the spectra spatial location and associating respectively the lower and higher score values to core and peripheral spatial location in the inset map. (e) Graph representing the spectral frequency distribution of PC3 scores in the tumour-associated cluster in the replica, with colours associating respectively the lower and higher score values to core and peripheral spatial location in the inset map, in the replica as well as observed in the section.

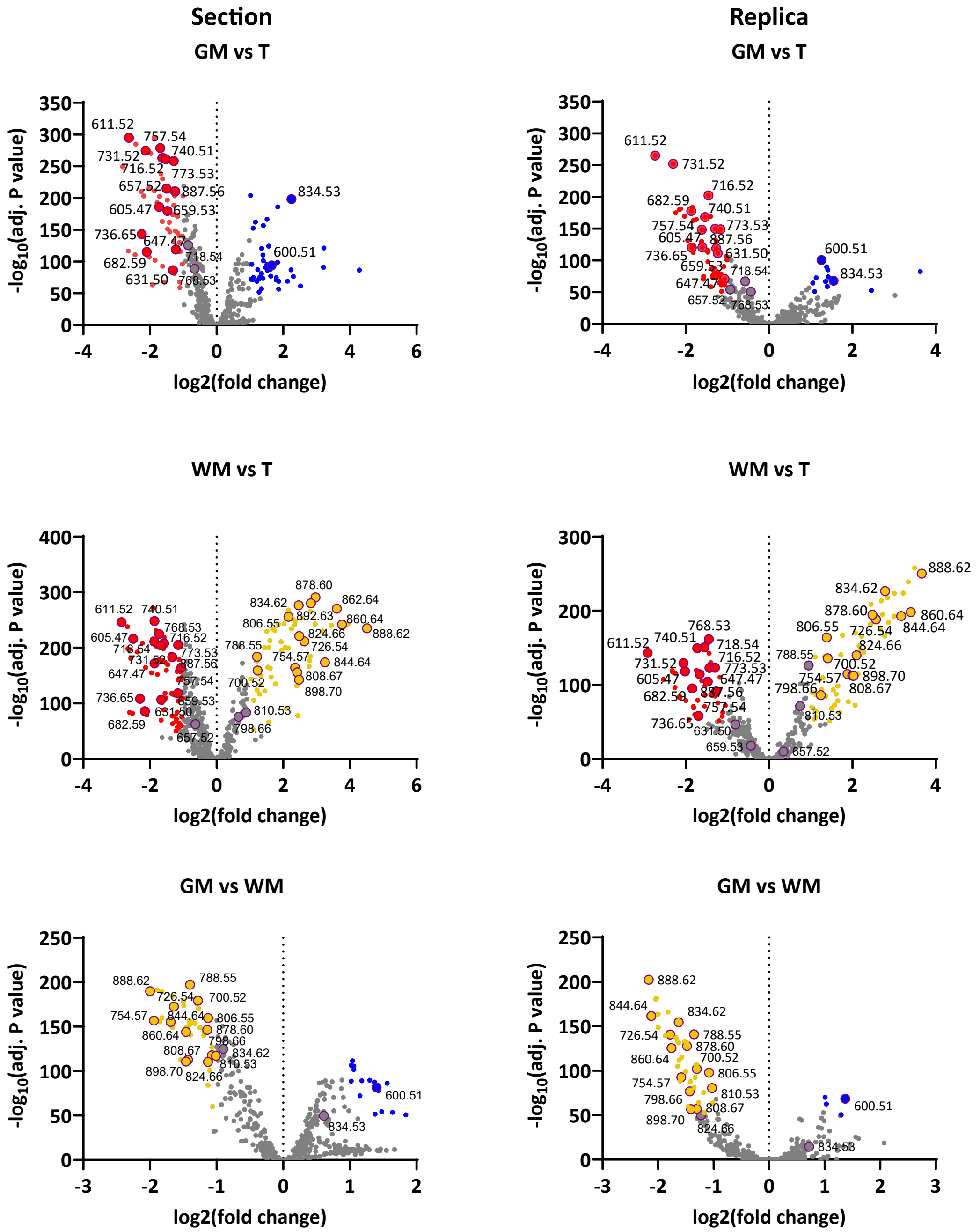

Figure S7: Differential lipid abundance across grey, white matter and tumour. Volcano plots showing the differential lipid distribution in pairwise comparisons between grey matter, white matter and tumour for section and replica, with highlighted representative lipid peaks. The volcano plots informed the selection of the 33 most representative peaks used in Fig. 4 i,j. which are labelled on the plots.

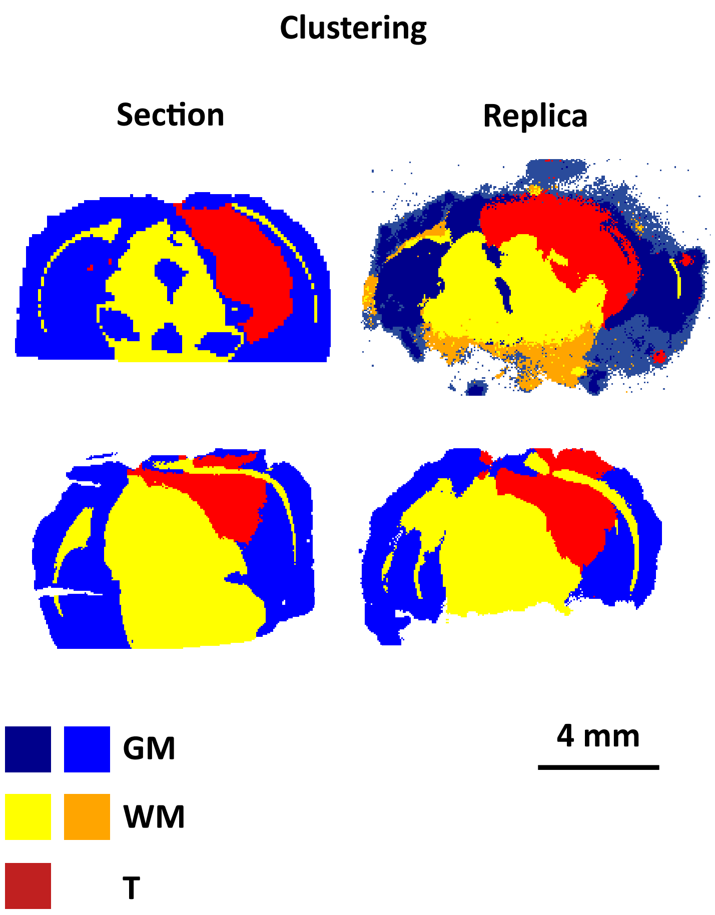

Figure S8: Hierarchical cluster analysis of tumour-bearing mouse brains. Maps of clusters associated to grey, white matter and tumour, obtained by HCA of DESI-MSI datasets of brain section and replica for two different tumour-bearing mice, supporting the reproducibility of the unsupervised classification of the tissue in section and replica.

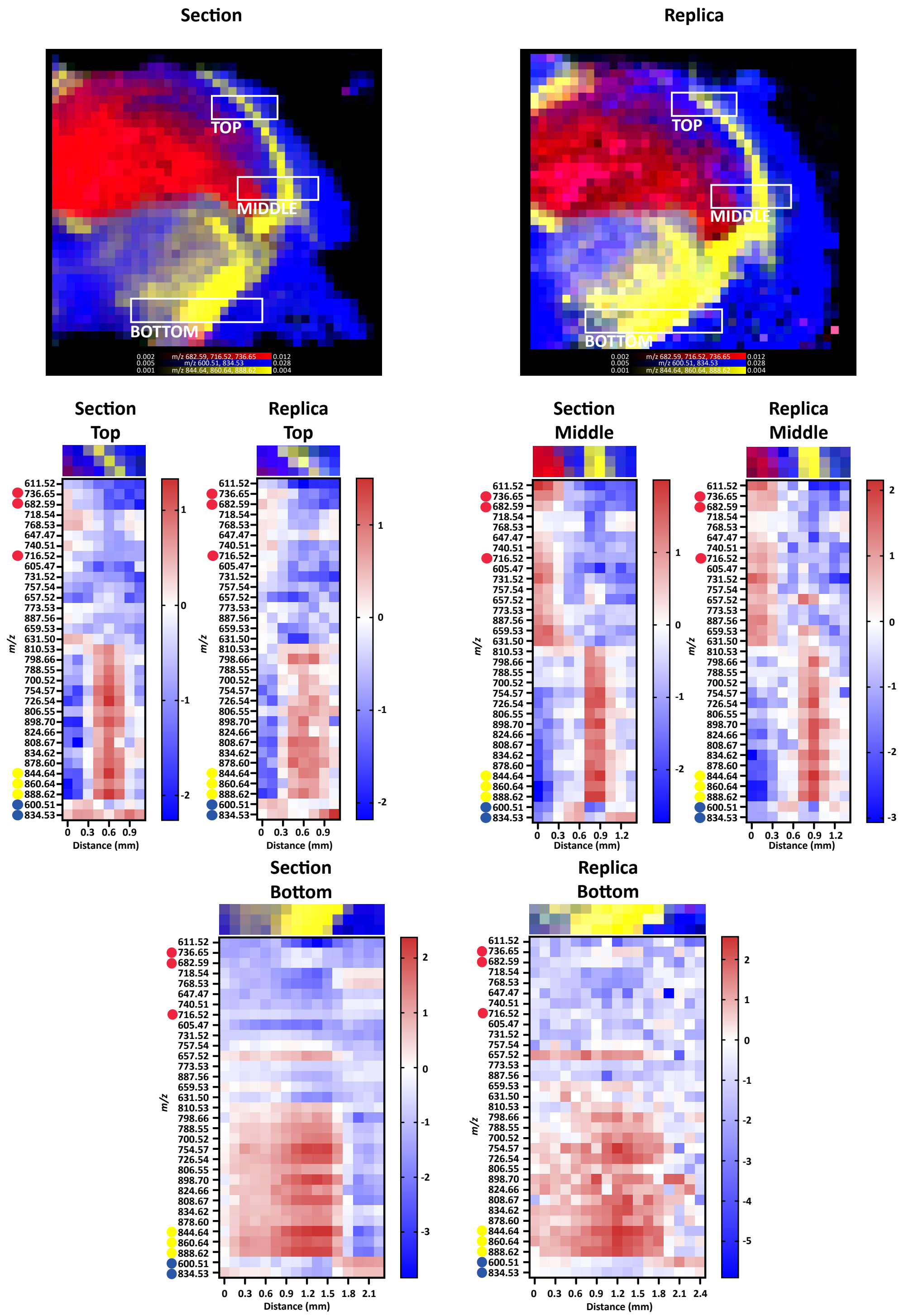

Figure S9: Comparison of spatial pattern and lipid abundance across multiple regions. Heatmaps showing the relative abundance along the major axis of the white boxes of the 33 lipid peaks identified by differential analysis within the section and the replica. Matching pattern and lipid abundance are shown in region of the corpus callosum near the tumour (TOP and MIDDLE) and in the region of the peduncle (BOTTOM).

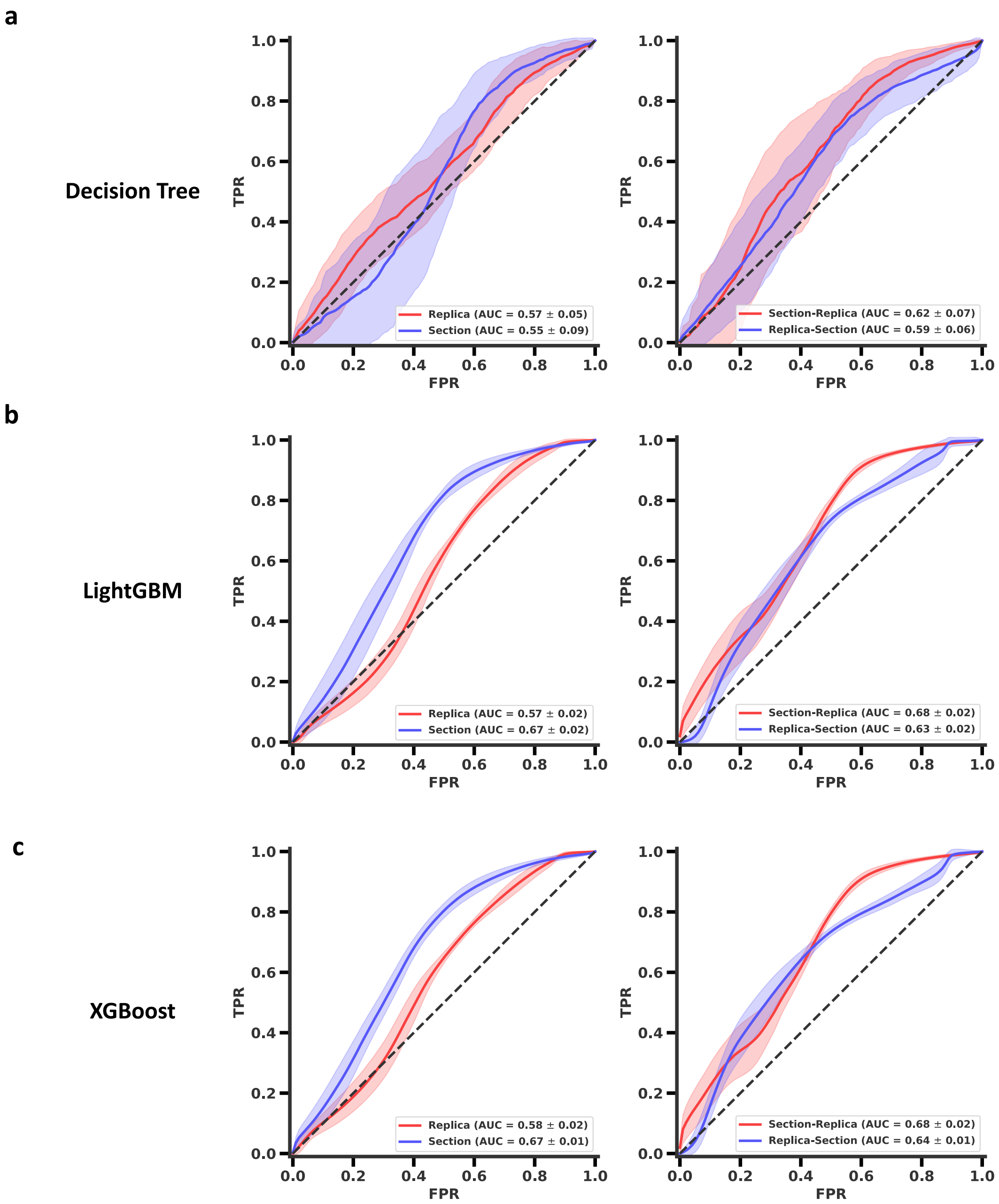

Figure S10: Receiver operator characteristics for the three additional models tested for tumor classification: Decision Tree, LightGBM and XGBoost. Data: mean with S.D. from 100 random-seed classifications.

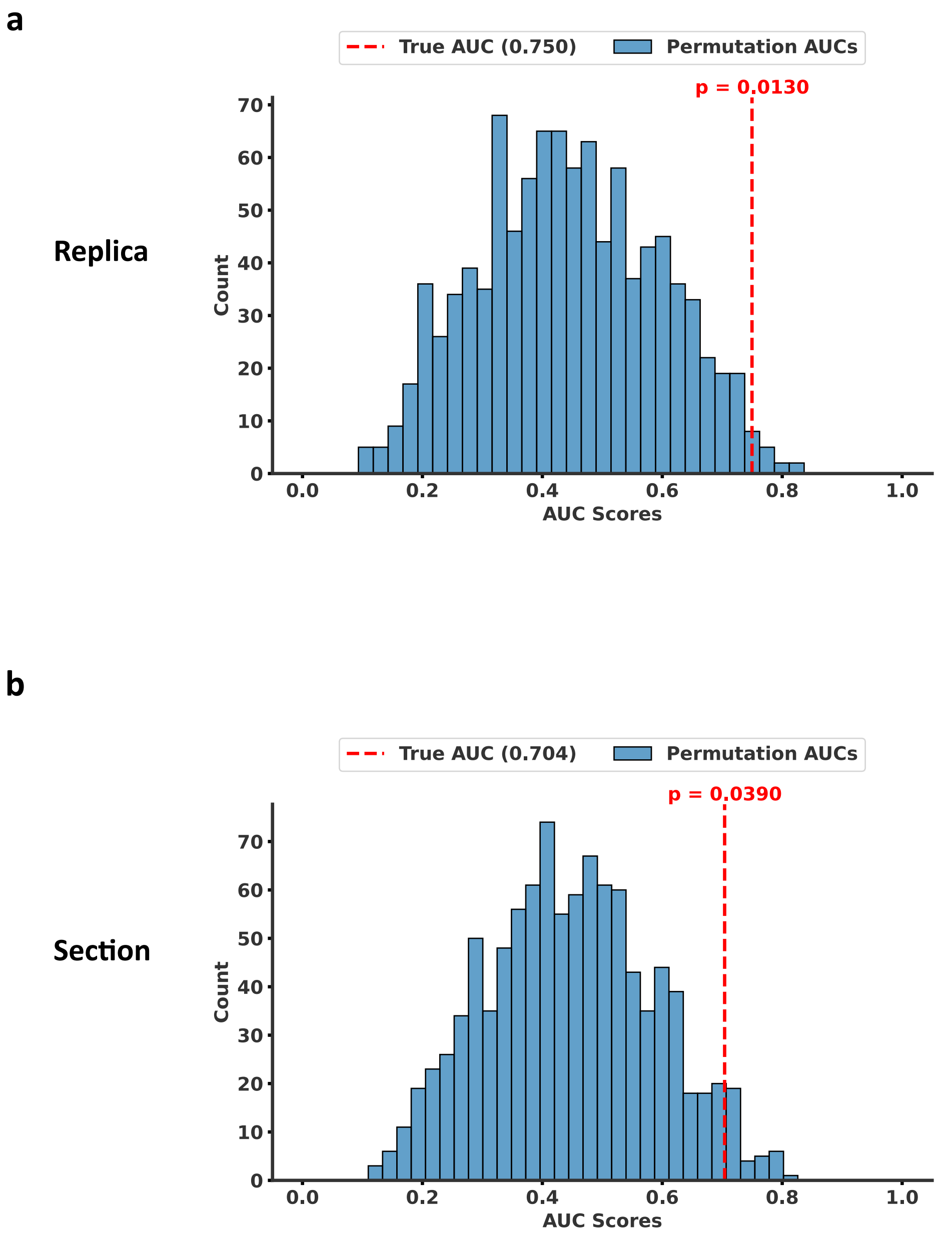

Figure S11: Logistic regression classifier permutation test distribution for AUC scores based on 1000 random-seed permutations of the samples grades for replicas (a) and sections (b). The true maximum AUC obtained from the 100 random-seed classifications indicated by the red dashed line.

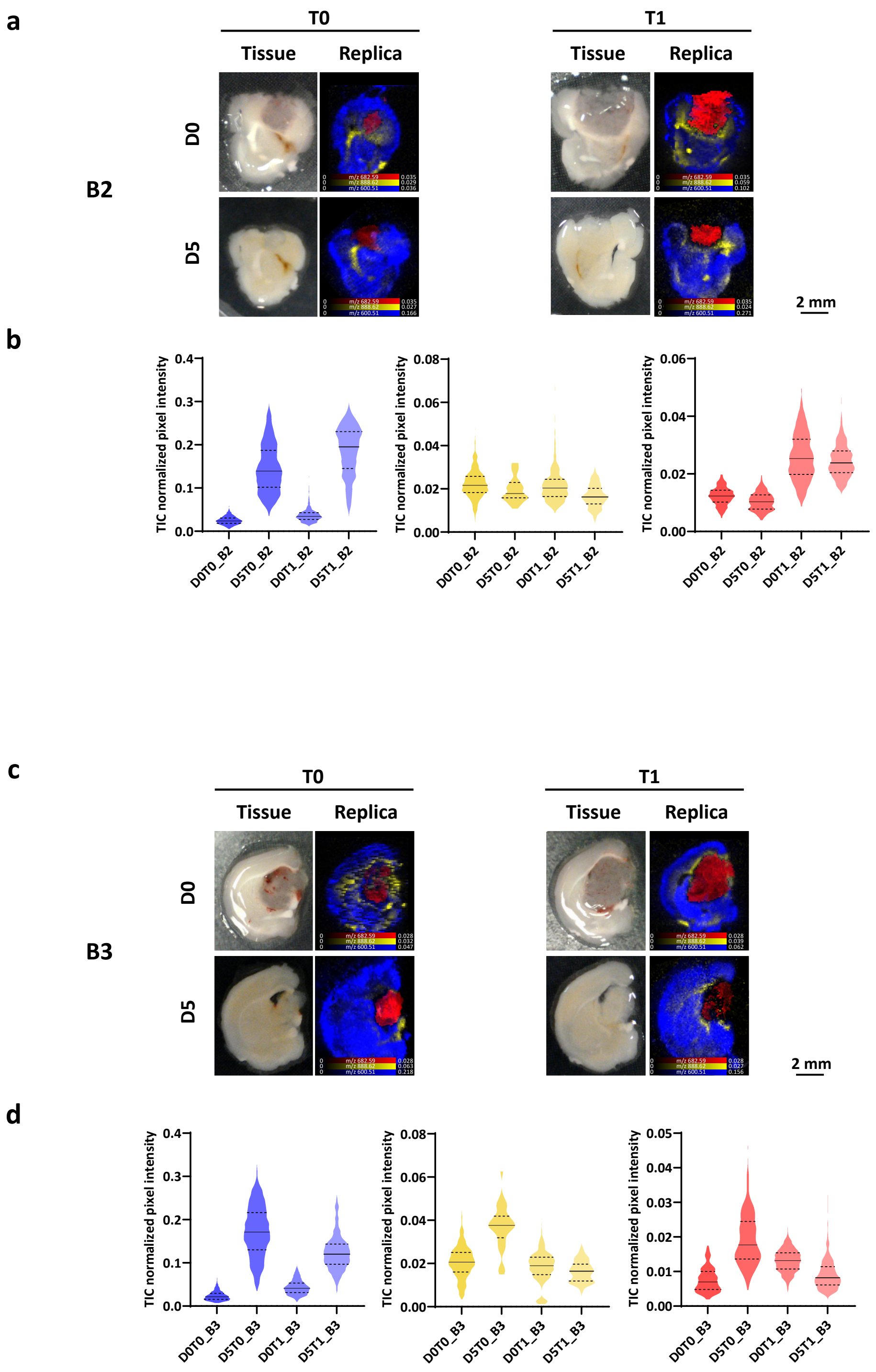

Figure S12: Spatiotemporal Lipidomics of molecular replicas generated from two additional tumour-bearing brains, showing (a) brain 2 (B2) tissue images and DESI imaging at day 0 (D0) and day 5 (D1), without treatment (T0) and with treatment (T1). (b) Violin plots showing the intensity distribution (TIC normalized) for the morphological markers corresponding to *m/z* values of 600.51 (GM, blue), 888.62 (WM, yellow), and 682.59 (tumor, red). Data: solid line: median; dashed lines: upper and lower quartiles. (c) brain 3 (B3) tissue images and DESI imaging at day 0 (D0) and day 5 (D1), without treatment (T0) and with treatment (T1), (d) Violin plots showing the intensity distribution (TIC normalized) for the morphological markers corresponding to *m/z* values of 600.51 (GM, blue), 888.62 (WM, yellow), and 682.59 (tumor, red). Data: solid line: median; dashed lines: upper and lower quartiles.

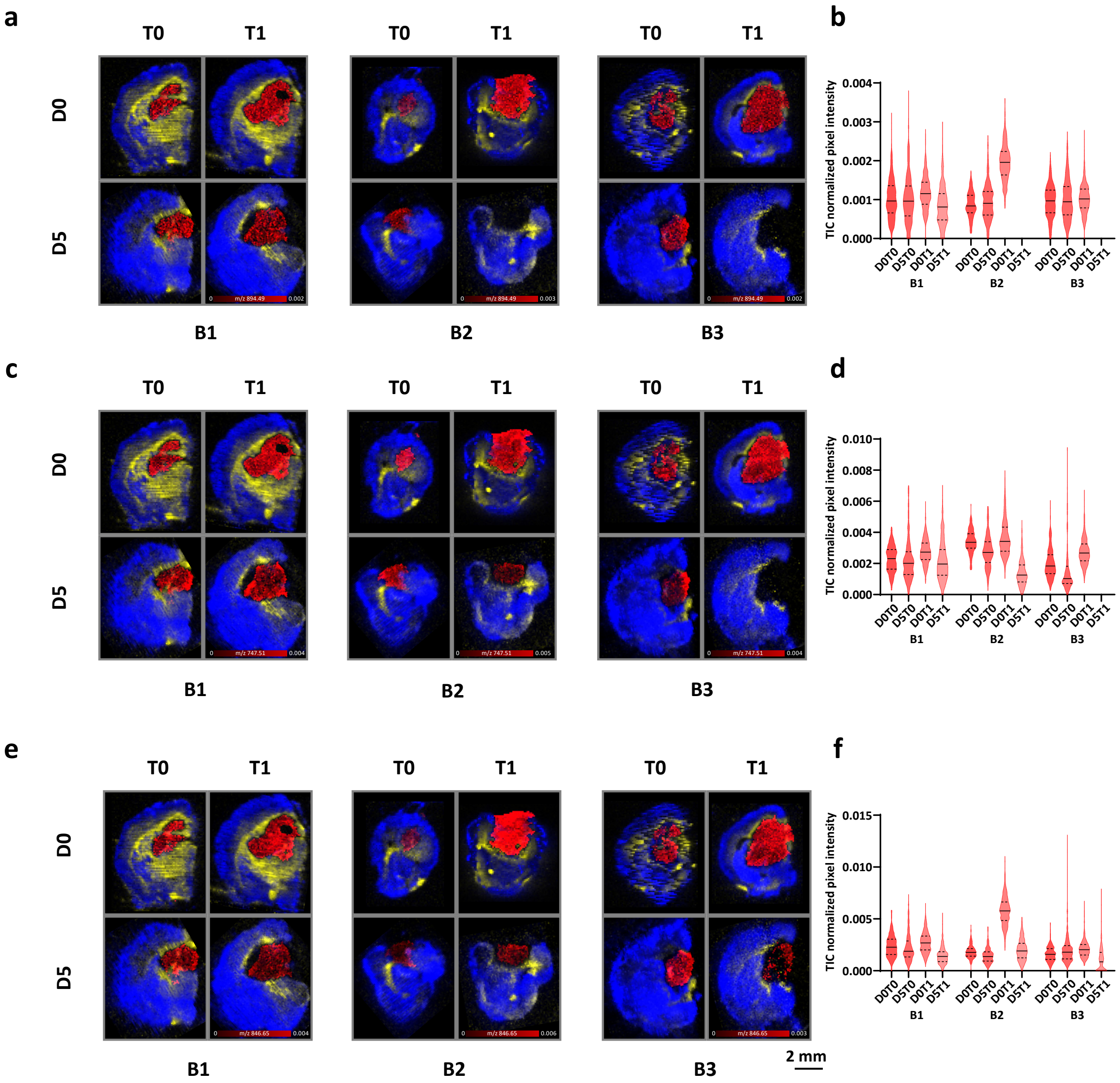

Figure S13: (a,c,e) DESI-MS images of lipids responding to TMZ treatment within the tumour and corresponding (b,d,f) violin plots of their intensity distribution within the tumour across brains and conditions. Data for *m/z* values of (a,b) 894.49, (c, d) 747.51, and (e, f) 846.65. (b,d,f) Data: solid line: median; dashed lines: upper and lower quartiles.

Table S1 Tandem mass spectrometry identification of lipids.

	Mass value*	Lipid name	Confidence	Matched frags	Reference mass value	Adduct	Cross-reference database	Reference
1	600.51	Cer 36:1;O2	4	4	565.54	[M+Cl]-	i, iii	[8,9]
2	680.54	Cer 43:0;O3	2	7	681.66	[M-H]-	i, ii	
3	681.59	DG 41:6	1	2	682.55	[M-H]-	ii, iv	
4	682.59	Cer 42:0;O4	2	3	683.64	[M-H]-	i, ii, iv	
5	692.45#	PS 29:0	1	0	693.46	[M-H]-	ii, iv	
6	700.52	PE O-34:2	3	4	701.54	[M-H]-	ii, iii, iv	[10,11]
7	716.52	PE 34:1	3	8	717.53	[M-H]-	i, ii, iii, iv	[12,13]
8	726.54	PE 35:3	3	3	727.52	[M-H]-	i, ii, iii, iv	[14]
9	727.52	PA 38:2	3	13	728.54	[M-H]-	ii, iii, iv	
10	736.65	Cer 46:1;O4	2	2	737.69	[M-H]-	ii	
11	740.51	PC 30:0	3	12	705.53	[M+Cl]-	i, ii, iii	[15]
12	747.51#	PG 34:1	3	17	748.53	[M-H]-	i, ii, iii, iv	[16,17]
13	788.55	PS 36:1	4	8	789.55	[M-H]-	i, ii, iii, iv	[12]
14	790.55	PE 40:6	4	10	791.55	[M-H]-	i, ii, iii, iv	[18–20]
15	810.52#	PS 38:4	4	7	811.54	[M-H]-	i, ii, iii, iv	[14,20]
16	834.53	PS 40:6	4	8	835.54	[M-H]-	i, iii, iv	[21,22]
17	844.64	PS O-41:1	3	3	845.65	[M-H]-	ii, iv	
18	846.65	PS O-41:0	3	2	847.67	[M-H]-	ii	
19	850.50	PE 42:8	1	13	815.55	[M+Cl]-	i, ii, iii, iv	
20	860.64	PS 41:0	4	9	861.65	[M-H]-	ii, iv	[14]
21	885.55	PI 38:4	3	19	886.56	[M-H]-	i, iii, iv	[12,21,23]
22	888.62	SHexCer 42:2;O2	3	6	889.63	[M-H]-	iii, iv	[24]
23	891.64#	PI O-39:1	2	2	892.64	[M-H]-	ii, iv	
24	894.49#	PS 42:8	4	5	859.54	[M+Cl]-	ii, iii, iv	[25]

*Mass value reported using the *m/z* value of 885.5498 for lock mass; #lipid identified by LC MS/MS; Reference databases: i: MassBank ; ii: LipidMaps; iii: HMDB , iv: SwissLipids

Table S2: Summary table of human glioma samples and their machine learning classification. The table indicates the unique biopsy number, the label used to identify each sample in the manuscript text and figures, whether the sample is a tissue section or a molecular replica, the histopathological annotations of the biopsy, the WHO grade and the results of the machine learning classification colour coded in green for correct assignments and orange for mis-assignments.

Biopsy	Sample Label	Sample Type	Histopathological Annotations	WHO Grade	ML Classification
1	HG 1-r	replica	oligodendroglioma, 1p19q codel	II	LOW
1	HG 1-s	section	oligodendroglioma, 1p19q codel	II	LOW
2	HG 2-r	replica	oligodendroglioma	II	LOW
2	HG 2-s	section	oligodendroglioma	II	HIGH
3	HG 3-r	replica	glioblastoma multiforme	IV	HIGH
3	HG 3-s	section	glioblastoma multiforme	IV	HIGH
4	HG 4-r	replica	glioblastoma multiforme	IV	HIGH
4	HG 4-s	section	glioblastoma multiforme	IV	HIGH
5	HG 5-r	replica	glioblastoma multiforme	IV	HIGH
5	HG 5-s	section	glioblastoma multiforme	IV	LOW
6	HG 6_1-r	replica	glioblastoma multiforme	IV	HIGH
6	HG 6_2-r	replica	glioblastoma multiforme	IV	HIGH
6	HG 6-s	section	glioblastoma multiforme	IV	HIGH
7	HG 7-r	replica	glioblastoma multiforme	IV	LOW
7	HG 7-s	section	glioblastoma multiforme	IV	LOW
8	HG 8-r	replica	anaplastic astrocytoma	III	HIGH
8	HG 8-s	section	anaplastic astrocytoma	III	HIGH
9	HG 9-r	replica	anaplastic oligodendroglioma, 1p19q codel	III	HIGH
9	HG 9-s	section	anaplastic oligodendroglioma, 1p19q codel	III	HIGH
10	HG 10-r	replica	secondary GBM – transf. of oligoastrocytoma	IV	HIGH
10	HG 10-s	section	secondary GBM – transf. of oligoastrocytoma	IV	HIGH
11	HG 11-r	replica	anaplastic astrocytoma	III	HIGH
11	HG 11_1-s	section	anaplastic astrocytoma	III	HIGH
11	HG 11_2-s	section	anaplastic astrocytoma	III	HIGH
12	HG 12-r	replica	glioblastoma multiforme	IV	HIGH
12	HG 12_1-s	section	glioblastoma multiforme	IV	HIGH
12	HG 12_2-s	section	glioblastoma multiforme	IV	HIGH
13	HG 13-r	replica	glioblastoma multiforme	IV	HIGH
13	HG 13-s	section	glioblastoma multiforme	IV	HIGH

14	HG 14-r	replica	glioblastoma multiforme	IV	HIGH
14	HG 14-s	section	glioblastoma multiforme	IV	HIGH
15	HG 15-r	replica	glioblastoma multiforme	IV	HIGH
15	HG 15-s	section	glioblastoma multiforme	IV	HIGH
16	HG 16-r	replica	pilocytic astrocytoma	I	HIGH
16	HG 16-s	section	pilocytic astrocytoma	I	HIGH
18	HG 18_1-r	replica	diffuse astrocytoma	II	HIGH
18	HG 18_2-r	replica	diffuse astrocytoma	II	HIGH
18	HG 18-s	section	diffuse astrocytoma	II	LOW
19	HG 19-r	replica	oligodendroglioma	II	HIGH
19	HG 19-s	section	oligodendroglioma	II	HIGH
20	HG 20-r	replica	diffuse astrocytoma, IDH1 mutant	II	LOW
20	HG 20-s	section	diffuse astrocytoma, IDH1 mutant	II	LOW
21	HG 21-r	replica	anaplastic oligodendroglioma, 1p19q codel, IDH1 mutant	III	LOW
21	HG 21-s	section	anaplastic oligodendroglioma, 1p19q codel, IDH1 mutant	III	LOW
23	HG 23-r	replica	pilocytic astrocytoma	I	LOW
23	HG 23-s	section	pilocytic astrocytoma	I	LOW
25	HG 25-r	replica	oligodendroglioma, 1p19q codel, IDH1 mutant	II	LOW
25	HG 25-s	section	oligodendroglioma, 1p19q codel, IDH1 mutant	II	LOW
29	HG 29-r	replica	oligodendroglioma, 1p19q codel	II	LOW
29	HG 29-s	section	oligodendroglioma, 1p19q codel	II	LOW

Table S3: Performance metrics for glioma classification. Table showing AUC and misclassified samples as a function of the combination of train and test dataset.

Train data biopsy type	Test data biopsy type	Mean AUC	STD AUC	Max AUC	Misclassified biopsies
section tissue	section tissue	0.71	0.05	0.804	HG 2-s, HG 5-s, HG 7-s, HG 16-s, HG 18-s, HG 19-s, HG 21-s
section tissue	replica tissue	0.7	0.05	0.800	HG 3-r, HG 4-r, HG 5-r, HG 7-r, HG 16-r, HG 19-r, HG 21-r, HG 25-r
replica tissue	replica tissue	0.75	0.02	0.801	HG 7-r, HG 16-r, HG 18_1-r, HG 18_2-r, HG 19-r, HG 21-r
replica tissue	section tissue	0.79	0.01	0.811	HG 7-s, HG 9-s, HG 11_1-s, HG 11_2-s, HG 16-s, HG 18-s, HG 19-s, HG 21-s

Table S4: leave one batch and patient out training process. Table showing number of train and test spectra as a function of the combination of train and test dataset.

Train samples	Test samples	Num. train spectras	Num. test spectras
HG 11_1-s, HG 11_2-s, HG 12_1-s, HG 14-s, HG 13-s, HG 15-s, HG 16-s, HG 19-s, HG 18-s, HG 20-s, HG 21-s, HG 23-s, HG 25-s, HG 29-s, HG 6-s, HG 7-s, HG 8-s, HG 12_2-s, HG 5-s, HG 4-s, HG 3-s, HG 2-s, HG 9-s, HG 10-s	HG 1-s	29909	1765
HG 14-s, HG 13-s, HG 15-s, HG 16-s, HG 19-s, HG 18-s, HG 20-s, HG 21-s, HG 23-s, HG 25-s, HG 29-s, HG 6-s, HG 7-s, HG 8-s, HG 5-s, HG 4-s, HG 3-s, HG 2-s, HG 9-s, HG 10-s, HG 1-s	HG 11_1-s, HG 11_2-s, HG 12_1-s, HG 12_2-s	25733	4432
HG 11_1-s, HG 11_2-s, HG 12_1-s, HG 15-s, HG 16-s, HG 19-s, HG 18-s, HG 20-s, HG 21-s, HG 23-s, HG 25-s, HG 29-s, HG 6-s, HG 7-s, HG 8-s, HG 12_2-s, HG 5-s, HG 4-s, HG 3-s, HG 2-s, HG 9-s, HG 10-s, HG 1-s	HG 14-s, HG 13-s	28431	3243
HG 11_1-s, HG 11_2-s, HG 12_1-s, HG 14-s, HG 13-s, HG 19-s, HG 18-s, HG 20-s, HG 21-s, HG 23-s, HG 25-s, HG 29-s, HG 6-s, HG 7-s, HG 8-s, HG 12_2-s, HG 5-s, HG 4-s, HG 3-s, HG 2-s, HG 9-s, HG 10-s, HG 1-s	HG 15-s, HG 16-s	29095	2579
HG 11_1-s, HG 11_2-s, HG 12_1-s, HG 14-s, HG 13-s, HG 15-s, HG 16-s, HG 20-s, HG 21-s, HG 23-s, HG 25-s, HG 29-s, HG 6-s, HG 7-s, HG 8-s, HG 12_2-s, HG 5-s, HG 4-s, HG 3-s, HG 2-s, HG 9-s, HG 10-s, HG 1-s	HG 19-s, HG 18-s	29698	1976
HG 11_1-s, HG 11_2-s, HG 12_1-s, HG 14-s, HG 13-s, HG 15-s, HG 16-s, HG 19-s, HG 18-s, HG 6-s, HG 7-s, HG 8-s, HG 12_2-s, HG 5-s, HG 4-s, HG 3-s, HG 2-s, HG 9-s, HG 10-s, HG 1-s	HG 20-s, HG 21-s, HG 23-s, HG 25-s, HG 29-s	23661	8013
HG 11_1-s, HG 11_2-s, HG 12_1-s, HG 14-s, HG 13-s, HG 15-s, HG 16-s, HG 19-s, HG 18-s, HG 20-s, HG 21-s, HG 23-s, HG 25-s, HG 29-s, HG 8-s, HG 12_2-s, HG 5-s, HG 4-s, HG 3-s, HG 2-s, HG 9-s, HG 10-s, HG 1-s	HG 6-s, HG 7-s	30273	1401
HG 11_1-s, HG 11_2-s, HG 14-s, HG 13-s, HG 15-s, HG 16-s, HG 19-s, HG 18-s, HG 20-s, HG 21-s, HG 23-s, HG 25-s, HG 29-s, HG 6-s, HG 7-s, HG 9-s, HG 10-s, HG 1-s	HG 12_1-s, HG 8-s, HG 12_2-s, HG 5-s, HG 4-s, HG 3-s, HG 2-s	23081	6629
HG 11_1-s, HG 11_2-s, HG 12_1-s, HG 14-s, HG 13-s, HG 15-s, HG 16-s, HG 19-s, HG 18-s, HG 20-s, HG 21-s, HG 23-s, HG 25-s, HG 29-s, HG 6-s, HG 7-s, HG 8-s, HG 12_2-s, HG 5-s, HG 4-s, HG 3-s, HG 2-s, HG 1-s	HG 9-s, HG 10-s	30038	1636
HG 12-r, HG 11-r, HG 14-r, HG 13-r, HG 16-r, HG 15-r, HG 18_1-r, HG 19-r, HG 18_2-r, HG	HG 1-r	41673	2556

20-r, HG 21-r, HG 23-r, HG 25-r, HG 29-r, HG 6_1-r, HG 6_2-r, HG 7-r, HG 8-r, HG 5-r, HG 4-r, HG 3-r, HG 2-r, HG 9-r, HG 10-r			
HG 14-r, HG 13-r, HG 16-r, HG 15-r, HG 18_1-r, HG 19-r, HG 18_2-r, HG 20-r, HG 21-r, HG 23-r, HG 25-r, HG 29-r, HG 6_1-r, HG 6_2-r, HG 7-r, HG 8-r, HG 5-r, HG 4-r, HG 3-r, HG 2-r, HG 9-r, HG 10-r, HG 1-r	HG 12-r, HG 11-r	41653	2576
HG 12-r, HG 11-r, HG 16-r, HG 15-r, HG 18_1-r, HG 19-r, HG 18_2-r, HG 20-r, HG 21-r, HG 23-r, HG 25-r, HG 29-r, HG 6_1-r, HG 6_2-r, HG 7-r, HG 8-r, HG 5-r, HG 4-r, HG 3-r, HG 2-r, HG 9-r, HG 10-r, HG 1-r	HG 14-r, HG 13-r	39178	5051
HG 12-r, HG 11-r, HG 14-r, HG 13-r, HG 18_1-r, HG 19-r, HG 18_2-r, HG 20-r, HG 21-r, HG 23-r, HG 25-r, HG 29-r, HG 6_1-r, HG 6_2-r, HG 7-r, HG 8-r, HG 5-r, HG 4-r, HG 3-r, HG 2-r, HG 9-r, HG 10-r, HG 1-r	HG 16-r, HG 15-r	41589	2640
HG 12-r, HG 11-r, HG 14-r, HG 13-r, HG 16-r, HG 15-r, HG 20-r, HG 21-r, HG 23-r, HG 25-r, HG 29-r, HG 6_1-r, HG 6_2-r, HG 7-r, HG 8-r, HG 5-r, HG 4-r, HG 3-r, HG 2-r, HG 9-r, HG 10-r, HG 1-r	HG 18_1-r, HG 19-r, HG 18_2-r	37898	6331
HG 12-r, HG 11-r, HG 14-r, HG 13-r, HG 16-r, HG 15-r, HG 18_1-r, HG 19-r, HG 18_2-r, HG 6_1-r, HG 6_2-r, HG 7-r, HG 8-r, HG 5-r, HG 4-r, HG 3-r, HG 2-r, HG 9-r, HG 10-r, HG 1-r	HG 20-r, HG 21-r, HG 23-r, HG 25-r, HG 29-r	34357	9872
HG 12-r, HG 11-r, HG 14-r, HG 13-r, HG 16-r, HG 15-r, HG 18_1-r, HG 19-r, HG 18_2-r, HG 20-r, HG 21-r, HG 23-r, HG 25-r, HG 29-r, HG 8-r, HG 5-r, HG 4-r, HG 3-r, HG 2-r, HG 9-r, HG 10-r, HG 1-r	HG 6_1-r, HG 6_2-r, HG 7-r	41073	3156
HG 12-r, HG 11-r, HG 14-r, HG 13-r, HG 16-r, HG 15-r, HG 18_1-r, HG 19-r, HG 18_2-r, HG 20-r, HG 21-r, HG 23-r, HG 25-r, HG 29-r, HG 6_1-r, HG 6_2-r, HG 7-r, HG 9-r, HG 10-r, HG 1-r	HG 8-r, HG 5-r, HG 4-r, HG 3-r, HG 2-r	36049	8180
HG 12-r, HG 11-r, HG 14-r, HG 13-r, HG 16-r, HG 15-r, HG 18_1-r, HG 19-r, HG 18_2-r, HG 20-r, HG 21-r, HG 23-r, HG 25-r, HG 29-r, HG 6_1-r, HG 6_2-r, HG 7-r, HG 8-r, HG 5-r, HG 4-r, HG 3-r, HG 2-r, HG 1-r	HG 9-r, HG 10-r	40362	3867

References

1. Eggers, L. & Schwudke, D. Lipid extraction: basics of the methyl-tert-butyl ether extraction. *Encyclopedia of lipidomics. Dordrecht: Springer Netherlands* 1–3 (2016).
2. Matyash, V., Liebisch, G., Kurzchalia, T. V., Shevchenko, A. & Schwudke, D. Lipid extraction by methyl-tert-butyl ether for high-throughput lipidomics. *J Lipid Res* **49**, 1137–1146 (2008).
3. Cajka, T. *et al.* Optimization of Mobile Phase Modifiers for Fast LC-MS-Based Untargeted Metabolomics and Lipidomics. *International Journal of Molecular Sciences* **24**, 1987 (2023).
4. Stoppini, L., Buchs, P.-A. & Muller, D. A simple method for organotypic cultures of nervous tissue. *Journal of Neuroscience Methods* **37**, 173–182 (1991).
5. Kim, H., Kim, E., Park, M., Lee, E. & Namkoong, K. Organotypic hippocampal slice culture from the adult mouse brain: A versatile tool for translational neuropsychopharmacology. *Progress in Neuro-Psychopharmacology and Biological Psychiatry* **41**, 36–43 (2013).
6. Jarmusch, A. K. *et al.* Differential lipid profiles of normal human brain matter and gliomas by positive and negative mode desorption electrospray ionization--mass spectrometry imaging. *PLoS One* **11**, e0163180 (2016).
7. Johno, H. *et al.* Detection of potential new biomarkers of atherosclerosis by probe electrospray ionization mass spectrometry. *Metabolomics* **14**, 38 (2018).
8. Jensen, M. *et al.* Opto-Lipidomics of Tissues. *Advanced Science* **11**, 2302962 (2024).
9. Tobias, F., Pathmasiri, K. C. & Cologna, S. M. Mass spectrometry imaging reveals ganglioside and ceramide localization patterns during cerebellar degeneration in the Npc1-/- mouse model. *Anal Bioanal Chem* **411**, 5659–5668 (2019).
10. Ventura, G., Bianco, M., Calvano, C. D., Losito, I. & Cataldi, T. R. I. Tandem Mass Spectrometry in Untargeted Lipidomics: A Case Study of Peripheral Blood Mononuclear Cells. *International Journal of Molecular Sciences* **25**, 12077 (2024).
11. Weir, J. M. *et al.* Plasma lipid profiling in a large population-based cohort [S]. *Journal of Lipid Research* **54**, 2898–2908 (2013).
12. Tsugawa, H. *et al.* Comprehensive identification of sphingolipid species by in silico retention time and tandem mass spectral library. *Journal of Cheminformatics* **9**, 19 (2017).
13. Kim, J. & Hoppel, C. L. Identification of unusual phospholipids from bovine heart mitochondria by HPLC-MS/MS. *Journal of Lipid Research* **61**, 1707–1719 (2020).
14. Edwards, G. *et al.* Endogenous ocular lipids as potential modulators of intraocular pressure. *Journal of Cellular and Molecular Medicine* **24**, 3856–3900 (2020).
15. Thi Le, T. T. *et al.* Lipid composition and molecular species of phospholipid in oyster Crassostrea lugubris (Sowerby, 1871) from Lang Co Beach, Hue Province, Vietnam. *Food Science & Nutrition* **9**, 4199–4210 (2021).
16. Calvano, C. D. *et al.* Analysis of Phospholipids, Lysophospholipids, and Their Linked Fatty Acyl Chains in Yellow Lupin Seeds (Lupinus luteus L.) by Liquid Chromatography and Tandem Mass Spectrometry. *Molecules* **25**, 805 (2020).
17. Claes, B. S. R. *et al.* Isomer-Resolved Mass Spectrometry Imaging of Acidic Phospholipids. *J. Am. Soc. Mass Spectrom.* **34**, 2269–2277 (2023).
18. Taware, R. *et al.* Lipidomics investigations into the tissue phospholipidomic landscape of invasive ductal carcinoma of the breast. *RSC Adv.* **11**, 397–407 (2020).
19. Oliveira-Lima, O. C. *et al.* Lipid dynamics in LPS-induced neuroinflammation by DESI-MS imaging. *Brain, Behavior, and Immunity* **79**, 186–194 (2019).
20. Zemski Berry, K. A., Gordon, W. C., Murphy, R. C. & Bazan, N. G. Spatial organization of lipids in the human retina and optic nerve by MALDI imaging mass spectrometry[S]. *Journal of Lipid Research* **55**, 504–515 (2014).
21. Janfelt, C., Wellner, N., Hansen, H. S. & Hansen, S. H. Displaced dual-mode imaging with desorption electrospray ionization for simultaneous mass spectrometry imaging in both polarities and with several scan modes. *Journal of Mass Spectrometry* **48**, 361–366 (2013).

22. Janfelt, C. *et al.* Visualization by mass spectrometry of 2-dimensional changes in rat brain lipids, including N-acylphosphatidylethanolamines, during neonatal brain ischemia. *The FASEB Journal* **26**, 2667–2673 (2012).
23. Chan, Y. H., Pathmasiri, K. C., Pierre-Jacques, D., Cologna, S. M. & Gao, R. Gel-assisted mass spectrometry imaging. *bioRxiv* 2023.06.02.543480 (2023) doi:10.1101/2023.06.02.543480.
24. Nielsen, M. M. B. *et al.* Mass spectrometry imaging of biomarker lipids for phagocytosis and signalling during focal cerebral ischaemia. *Sci Rep* **6**, 39571 (2016).
25. Fruehwirth, S. *et al.* In Vitro Digestion of Grape Seed Oil Inhibits Phospholipid-Regulating Effects of Oxidized Lipids. *Biomolecules* **10**, 708 (2020).
