# Supplementary material for: Nanoneedles enable spatiotemporal lipidomics of living tissues
Source: Nat Nanotechnol. 2025 Jun 16;20(9):1262–72. doi: 10.1038/s41565-025-01955-8 (PMC12443637; doi:10.1038/s41565-025-01955-8)
Supplement: Supplementary file 2 — Reporting Summary [file 41565_2025_1955_MOESM2_ESM.pdf]

nature portfolio

Corresponding author(s): Ciro Chiappini

Last updated by author(s): 2025/22/04

Reporting Summary

Nature Portfolio wishes to improve the reproducibility of the work that we publish. This form provides structure for consistency and transparency in reporting. For further information on Nature Portfolio policies, see our Editorial Policies and the Editorial Policy Checklist.

Statistics

For all statistical analyses, confirm that the following items are present in the figure legend, table legend, main text, or Methods section.

n/a	Confirmed	
☐	☒	The exact sample size (*n*) for each experimental group/condition, given as a discrete number and unit of measurement
☐	☒	A statement on whether measurements were taken from distinct samples or whether the same sample was measured repeatedly
☐	☒	The statistical test(s) used AND whether they are one- or two-sided<br>*Only common tests should be described solely by name; describe more complex techniques in the Methods section.*
☐	☒	A description of all covariates tested
☐	☒	A description of any assumptions or corrections, such as tests of normality and adjustment for multiple comparisons
☐	☒	A full description of the statistical parameters including central tendency (e.g. means) or other basic estimates (e.g. regression coefficient) AND variation (e.g. standard deviation) or associated estimates of uncertainty (e.g. confidence intervals)
☐	☒	For null hypothesis testing, the test statistic (e.g. *F*, *t*, *r*) with confidence intervals, effect sizes, degrees of freedom and *P* value noted<br>*Give P values as exact values whenever suitable.*
☒	☐	For Bayesian analysis, information on the choice of priors and Markov chain Monte Carlo settings
☐	☒	For hierarchical and complex designs, identification of the appropriate level for tests and full reporting of outcomes
☐	☒	Estimates of effect sizes (e.g. Cohen's *d*, Pearson's *r*), indicating how they were calculated

Our web collection on statistics for biologists contains articles on many of the points above.

Software and code

Policy information about availability of computer code

Data collection	ESI-MS, DESI-MSI and DESI MS/MS data were collected using MassLynx 4.2 and HD Imaging 1.4. LC-MS/MS data were collected using Thermo Xcalibur 3.0.63. SEM images were collected using ZEISS SmartSEM. Widefield fluorescence microscopy images were collected using Leica Application Suite X 3.7.5. Confocal fluorescence microscopy images were collected using Zeiss Zen blue version 3.5. 5.093. The finite element simulations were performed using COMSOL Multiphysics® 5.4. Quantification of biomolecules were collected using CLARIOstar® Plus microplate reader software version 5.21.
Data analysis	MS/MS data for lipid identification was performed using LipostarMSI version 2.1.0b3.<br>Unbiased DESI-MSI data processing was performed using Python 3.11 (https://github.com/zaritskylab/Nanoneedle-Lipidomics).<br>Common representation of lipids were generated by HD Imaging 1.4, and analyzed using R version 4.1.2 and MATLAB 2020a (https://github.com/ChiappiniLab/Nanoneedle-Lipidomics).<br>Statistical analysis and graphical data visualization was performed using GraphPad Prism 9 and 10.

For manuscripts utilizing custom algorithms or software that are central to the research but not yet described in published literature, software must be made available to editors and reviewers. We strongly encourage code deposition in a community repository (e.g. GitHub). See the Nature Portfolio guidelines for submitting code & software for further information.

Data

Policy information about availability of data

All manuscripts must include a data availability statement. This statement should provide the following information, where applicable:

- Accession codes, unique identifiers, or web links for publicly available datasets
- A description of any restrictions on data availability
- For clinical datasets or third party data, please ensure that the statement adheres to our policy

All data presented in the manuscript including all mass spectrometry datasets are available from the KORDS repository http://doi.org/10.18742/c.7711667

Research involving human participants, their data, or biological material

Policy information about studies with human participants or human data. See also policy information about sex, gender (identity/presentation), and sexual orientation and race, ethnicity and racism.

Reporting on sex and gender	Sex and gender was removed as part of the anonymisation process prior to accessing the samples, and thus was not available to the authors.
Reporting on race, ethnicity, or other socially relevant groupings	any background data was removed as part of the anonymisation process prior to accessing the samples, and thus was not available to the authors.
Population characteristics	any characteristic data was removed as part of the anonymisation process prior to accessing the samples, and thus was not available to the authors.
Recruitment	Human brain tissue samples were obtained as surplus material during glioma surgeries conducted at NHS Lothian. All participants provided informed consent for the use of their tissue in research, in accordance with the ethical approval granted by the relevant NHS Research Ethics Committee. Only samples deemed excess to diagnostic requirements were included in the study.
Ethics oversight	Lothian NRS Bioresource 20/ES/0061

Note that full information on the approval of the study protocol must also be provided in the manuscript.

Field-specific reporting

Please select the one below that is the best fit for your research. If you are not sure, read the appropriate sections before making your selection.

☒ Life sciences ☐ Behavioural & social sciences ☐ Ecological, evolutionary & environmental sciences

For a reference copy of the document with all sections, see nature.com/documents/nr-reporting-summary-flat.pdf

Life sciences study design

All studies must disclose on these points even when the disclosure is negative.

Sample size	No statistical methods were used to pre-determine sample sizes but our sample sizes are similar to those reported in previous publications
Data exclusions	no data were excluded
Replication	All experiments presented were reproduced at least three times using distinct samples.
Randomization	Samples were randomly selected for assignment to the experimental groups to ensure unbiased distribution across conditions. Experimental conditions and stimulus presentation were also organized without any systematic bias to ensure that each sample had an equal chance of being exposed to any given condition.
Blinding	Data collection and analysis were not performed blind to the conditions of the experiments

Reporting for specific materials, systems and methods

We require information from authors about some types of materials, experimental systems and methods used in many studies. Here, indicate whether each material, system or method listed is relevant to your study. If you are not sure if a list item applies to your research, read the appropriate section before selecting a response.

Materials & experimental systems

n/a	Involved in the study
☒	☐ Antibodies
☒	☐ Eukaryotic cell lines
☒	☐ Palaeontology and archaeology
☐	☒ Animals and other organisms
☒	☐ Clinical data
☒	☐ Dual use research of concern
☒	☐ Plants

Methods

n/a	Involved in the study
☒	☐ ChIP-seq
☒	☐ Flow cytometry
☒	☐ MRI-based neuroimaging

Animals and other research organisms

Policy information about studies involving animals; ARRIVE guidelines recommended for reporting animal research, and Sex and Gender in Research

Laboratory animals	animal used included adult CD1 mice and C57BL/6j, injected with neural stem cells with concomitant Nf1, Pten, EGFR-vIII mutations
Wild animals	no wild animals included
Reporting on sex	sex information was not collected.
Field-collected samples	no field-collected samples
Ethics oversight	UK Home Office project license PBE6EB195

Note that full information on the approval of the study protocol must also be provided in the manuscript.

Plants

Seed stocks	n/a
Novel plant genotypes	n/a
Authentication	n.a
